# SGLT2 Inhibitors in Cardio-Oncology: A Systematic Review and Meta-Analysis

**DOI:** 10.1016/j.jacadv.2026.102790

**Published:** 2026-05-14

**Authors:** Luigi Spadafora, Marco Bernardi, Gianmarco Sarto, Beatrice Simeone, Erica Rocco, Salvatore Carbone, Laura Adelaide Dalla Vecchia, Roberto Franco Enrico Pedretti, Valentina Valenti, Rachele Di Mario, Maurizio Forte, Giacomo Frati, Antonio Abbate, Michele Golino, Francesco Versaci, Mariangela Peruzzi, Pierre Sabouret, Giuseppe Biondi Zoccai, Sebastiano Sciarretta

**Affiliations:** a Department of Medical-Surgical Sciences and Biotechnologies, Sapienza University of Rome, Latina, Italy; b UOC UTIC Emodinamica e Cardiologia, Santa Maria Goretti Hospital, Latina, Italy; c Cardiology Division, ICOT Istituto ‘Marco Pasquali' University Hospital, Latina, Italy; d Nutrition Program, EVMS School of Health Professions, Macon & Joan Brock Virginia Health Sciences at Old Dominion University, Norfolk, Virginia; e Division of Endocrine and Metabolic Disorders, Strelitz Diabetes Center, Department of Medicine, Eastern Virginia Medical School, Macon & Joan Brock Virginia Health Sciences at Old Dominion University, Norfolk, Virginia; f Department of Cardiology, IRCCS Istituti Clinici Scientifici Maugeri, Milan, Italy; g School of Medicine and Surgery, University of Milano-Bicocca, Milan, Italy; h Cardiology Unit, Hospital of Erba, Erba, Italy; i Maria Cecilia Hospital, GVM Care and Research, Cotignola, Italy; j IRCCS NeuroMed, Pozzilli, Italy; k Berne Cardiovascular Research Center, University of Virginia, Charlottesville, Virginia, USA; l Division of Cardiology, University of Florida College of Medicine - Jacksonville, Jacksonville, Florida, USA; m Pauley Heart Center, Division of Cardiology, Virginia Commonwealth University, Richmond, Virginia, USA; n Department of Medical and Cardiovascular Sciences, “Sapienza” University of Rome, Rome, Italy; o Cardiology Department, Institut de Cardiologie, Centre Hospitalier Universitaire La Pitié-Salpêtrière, Sorbonne Université, Paris, France

**Keywords:** cardio-oncology, heart failure, sodium-glucose cotransporter 2 inhibitors

## Abstract

**Background:**

According to available evidence, sodium-glucose cotransporter-2 inhibitors (SGLT2i) may confer cardioprotection in patients with cancer undergoing chemotherapy.

**Objectives:**

The objective of the study was to evaluate the impact of SGLT2i on all-cause mortality and heart failure (HF) outcomes in this population.

**Methods:**

We searched PubMed, Cochrane CENTRAL, and Embase through August 2025 for studies of SGLT2i in adult patients with cancer. Random-effects models were used to pool effects for all-cause mortality and an HF composite (new-onset HF and/or HF hospitalization) in cancer patients >18 years. Meta-regression tested effect modification by beta-blockers, statins, angiotensin-converting enzyme inhibitors/angiotensin receptor blockers, age, and sex.

**Results:**

Thirteen observational studies were included, for a total of 107,126 patients. The median age was 67.6 years (IQR: 62.5-71.0); median follow-up was 24 months (IQR: 19.2-29.0), nearly all patients had diabetes mellitus. SGLT2i were associated with lower all-cause mortality (risk ratio [RR]: 0.47; 95% CI: 0.38-0.58; I^2^ = 96.30%; *P* < 0.001), with no effect modification by background therapies, age, or sex (all *P* > 0.05). The HF composite was also significantly reduced (RR: 0.48; 95% CI: 0.29-0.78; I^2^ = 87.01%), again with no evidence that background therapies modified the effect (all *P* > 0.05). Risk of atrial fibrillation/atrial flutter was lower with SGLT2i (RR: 0.57; 95% CI: 0.42-0.76; I^2^ = 66.4%; *P* < 0.001). Safety outcomes were not increased in the SGLT2i arm.

**Conclusions:**

In patients with cancer, SGLT2i appear safe and are associated with fewer deaths and HF events, although substantial heterogeneity was observed across studies. Randomized controlled trials are warranted to confirm these hypothesis-generating findings.

Sodium-glucose cotransporter 2 inhibitors (SGLT2i) have become a cornerstone of care for heart failure (HF), chronic kidney disease, and type 2 diabetes mellitus (T2DM).^1^^,^^2^ Their cardiovascular benefits extend beyond glycosuria and include natriuresis and hemodynamic unloading, improvement in cellular bioenergetics and mitochondrial function, and attenuation of oxidative stress.^2^ Many of these pathways are directly implicated in cancer therapy–related cardiac dysfunction, which is an increasingly common clinical problem.^3^ For instance, it is estimated that anthracyclines are a major cause of both acute and chronic cardiotoxicity, and long-term cardiovascular sequelae account for roughly one-third of deaths among cancer survivors.^4^^,^^5^ In a cohort of 2,625 patients treated with anthracyclines and followed up for a median of 5.2 years, 9% developed cardiotoxicity, with the highest incidence occurring in the first year after therapy completion.^6^ On this background, several preclinical and clinical studies evaluated SGLT2i for the prevention or treatment of cancer therapy–related cardiac dysfunction, with evidence supporting the reduction of all-cause mortality and HF events.^7^, ^8^, ^9^, ^10^, ^11^, ^12^, ^13^, ^14^, ^15^, ^16^, ^17^, ^18^, ^19^ Recent meta-analyses of observational cohorts support these favorable associations but did not assess whether the apparent benefits vary according to concomitant cardioprotective medications, age, or sex.^20^, ^21^, ^22^ Current cardio-oncology guidelines highlight a range of cardioprotective strategies for high-risk patients undergoing potentially cardiotoxic regimens, including beta-blockers, angiotensin-converting enzyme inhibitors (ACEIs) or angiotensin receptor blockers (ARBs), statins, and dexrazoxane, while also calling for new approaches to broaden the therapeutic armamentarium.^23^ To fill this evidence gap, we conducted an updated systematic review and meta-analysis, including prespecified study-level meta-regressions, to quantify the association between SGLT2i use and mortality or HF outcomes in patients with cancer, and to explore whether these associations vary according to the prevalence of concomitant cardioprotective therapies, age, or sex.

## Methods

We conducted a Preferred Reporting Items for Systematic Reviews and Meta-Analyses (PRISMA)-conforming systematic review and meta-analysis (PROSPERO: CRD420250653585) of observational studies and randomized trials published up to August 2025 that evaluated SGLT2i in adults (≥18 years) with cancer and reported cardiovascular outcomes. Methodological adaptations were implemented during the conduct of the study to better reflect the available evidence and enhance the robustness and interpretability of the analyses. Eligible studies enrolled patients with any tumor type; exposure could be any SGLT2 inhibitor; at minimum, all-cause mortality had to be reported as an outcome and a placebo or active comparator arm was required. This study was based exclusively on data extracted from previously published studies. No individual-level identifiable patient data were collected or accessed. Therefore, Institutional Review Board approval and informed consent were not required.

### Search strategy and data extraction

We included peer-reviewed, English-language articles and excluded conference abstracts and nonpeer-reviewed reports. We searched PubMed, Cochrane CENTRAL, and Embase using the terms “sodium glucose cotransporter 2 inhibitors,” “SGLT2 inhibitors,” “cancer,” “cardio-oncology,” and “cardiotoxicity,” and we screened reference lists of included studies and prior syntheses (Supplemental Table 1). Two reviewers independently screened titles/abstracts and full texts, extracted data in duplicate with a piloted form (study characteristics, follow-up, crude events and denominators by arm, and study-level covariates including mean age, male, hypertension, dyslipidemia, diabetes, chronic kidney disease, smoking, cancer class, baseline left ventricular ejection fraction (LVEF), and background beta-blocker, statin, and ACEI/ARB use, and resolved disagreements with a third reviewer. All included studies were observational; risk of bias was assessed independently by 2 reviewers using ROBINS-I (overall judgment: low, moderate, serious, or critical).

### Endpoints

Primary endpoints were all-cause mortality and an HF composite, defined as new-onset HF and/or HF hospitalization according to study-specific definitions. When both components were available, they were jointly considered to capture the overall burden of HF events; otherwise, the available definition was retained. A sensitivity analysis focusing on HF hospitalization only was also performed. Secondary endpoints were a composite of atrial fibrillation or atrial flutter, myocardial infarction, urinary tract infection (UTI), sepsis, stroke, and acute kidney injury (AKI). Endpoint definitions are further detailed in Supplemental Table 2.

### Statistical analysis

We calculated crude risk ratios (RRs) and their 95% CIs from each study by reconstructing 2 × 2 tables whenever possible. We chose to rely on crude estimates because adjusted HRs or ORs were not consistently reported across studies, and using crude data ensured comparability across the evidence base, in line with previous meta-analyses on this topic.^20^^,^^21^ Random-effects models with restricted maximum likelihood (REML) were specified a priori because we expected genuine between-study variability in effects due to differences in tumor types and staging, oncologic regimens (eg, anthracyclines, anti-human epidermal growth factor receptor 2 [anti-HER2] agents, tyrosine kinase inhibitors [TKIs], and immune checkpoint inhibitors [ICIs]), baseline cardiovascular risk, background cardioprotective therapy, follow-up duration, and SGLT2i agent/dose—conditions under which a common-effect assumption is unlikely. We summarized heterogeneity with τ^2^, I^2^, and Cochran’s Q (with *P* for heterogeneity), and we reported 2-sided z-tests with 95% CIs for pooled effect sizes. Small-study effects were explored visually with funnel plots and formally with the Egger test, interpreted cautiously particularly when fewer than 10 studies were available. In addition to funnel plots and Egger test, small-study effects were further explored using Doi plots and the Luis Furuya-Kanamori (LFK) index. Study-level meta-regression analyses were conducted using the meta-reg command in Stata 18, applying a random-effects model with REML estimation and Knapp-Hartung adjustment for SEs. Given the limited number of studies, only univariable meta-regressions were performed to explore the potential influence of individual moderators (ie, prevalence of beta-blocker, ACEI/ARB, and statin therapy) on the effect estimates, thereby avoiding model overfitting and unstable coefficients. Meta-regression analyses were restricted to covariates available in the majority of included studies. All analyses were conducted in Stata (version 18.0; StataCorp LLC).

### Sensitivity analyses

As a sensitivity analysis, we repeated the meta-analysis, focusing on our key primary endpoints, including only cohorts in which all participants had received at least 1 cycle of anthracyclines. To further explore potential sources of heterogeneity, we performed prespecified study-level meta-regressions assessing whether the associations between SGLT2i use and outcomes were influenced by clinical or methodological moderators, including tumor type, cardiovascular risk factors, baseline LVEF, and follow-up duration. Furthermore, we conducted a separate meta-analysis restricted to studies reporting confounding-adjusted effect estimates (HRs) for all-cause mortality. Comparable adjusted estimates were not consistently available for other outcomes across the included studies and were therefore not pooled. Additional sensitivity analyses accounted for potential cohort overlap and restricted outcomes to HF hospitalization-only events.

## Results

### Baseline features of the population

Thirteen observational studies were included, for a total of 107,126 patients, of whom 30,612 were treated with SGLT2i and 76,514 served as controls (Supplemental Tables 3 and 4.1). We deliberately excluded conference abstracts to ensure robustness of the evidence base. In addition, the study by Abbas et al. was excluded because crude data on mortality were not reported, whereas for the endpoint of all-cause mortality, we excluded Daniele et al.8 as the outcome was defined as a composite of death or hospitalization for HF rather than mortality alone.^8^, ^24^ Based on study-level data, we estimate that approximately 103,000 patients were enrolled in studies involving active cancer phases. Cancer activity status was not clearly specified in a minority of studies, particularly Avula et al. and Henson et al. Further details are provided in Supplemental Tables 4.1 and 4.2. The timing of SGLT2i initiation relative to cancer therapy was heterogeneous across studies. In most cohorts, SGLT2i use occurred concomitantly with anticancer treatment, particularly in studies including patients receiving chemotherapy or immune checkpoint inhibitors (Supplemental Tables 4.1 and 4.2). However, these data were reported at the study level, and information regarding continuation of SGLT2i therapy after completion of cancer treatment was generally not available. A detailed description of SGLT2i exposure definitions across studies is provided in Supplemental Table 4.2. T2DM was the predominant condition across included studies. Although most studies explicitly specified T2DM, some cohorts reported diabetes more generally without distinguishing subtypes (eg, Abdel-Qadir et al., Fath et al., Gongora et al.), and diabetes subtype was not specified in Henson et al. Notably, type 1 diabetes mellitus was not mentioned in any of the included studies. Details are provided in Supplemental Table 4.2. Reporting of race and ethnicity was variable across studies. Among those reporting this information, White race was generally predominant, whereas Black and other racial or ethnic groups were poorly represented. Two studies did not report race/ethnicity (Abdel-Qadir et al., Perelman et al.), and several studies conducted in Asian populations did not provide detailed breakdowns (Supplemental Tables 4.1 and 4.2). The specific type of SGLT2i was reported in few studies. When available, empagliflozin was the most frequently used agent (eg, Chiang et al., Fath et al., Gongora et al., Perelman et al.), whereas canagliflozin predominated in Luo et al. (Supplemental Tables 4.1 and 4.2). Across studies, the median age was 67.6 years (IQR: 62.5-71.0) and approximately half of the patients were male (Supplemental Tables 4.1 and 4.2). Follow-up was typically long enough to capture medium-term outcomes (median 24.0 months; IQR: 19.2-29.0). Baseline LVEF was reported in 5 studies and centered in the preserved range (median 58.8%; IQR: 53.5-63.0). Breast cancer (median 25.5%; IQR: 17.5-33.9), gastrointestinal malignancies (19.4%; IQR: 16.6-62.0), and hematologic malignancies (33.4%; IQR: 19.2-36.2) were most frequently represented among studies reporting tumor class. With regard to cardiovascular risk factors and comorbidities, hypertension was present in a median of 75.2% (IQR: 62.0-93.2), dyslipidemia in 59.0% (IQR: 52.5-64.9; 8/13), chronic kidney disease in 21.0% (IQR: 11.0-42.2; 9/13). Concomitant cardioprotective therapies were commonly used: beta-blockers were prescribed in a median of 47.5% (IQR: 31.9-55.1; 10/13 studies); statins in 69.8% (IQR: 61.0-72.2; 10/13); and ACEI or ARBs in 50.7% (IQR: 47.0-73.0; 10/13). Primary prevention HF cohorts (no HF at baseline) were explicitly identified in 8 studies. Anthracycline exposure was frequent across cohorts (5 studies, for a total of 8,235 patients); where cumulative dose was reported, the median was 252.0 mg/m^2^ (IQR: 222.8-281.2). Beyond anthracycline-based regimens, patients were exposed to a broad range of anticancer therapies, including antimetabolites, alkylating agents, platinum, and antimicrotubule agents. Targeted therapies (eg, anti-vascular endothelial growth factor [anti-VEGF] and anti-HER2 agents) and, in selected cohorts, immune checkpoint inhibitors were also represented, either alone or in combination with chemotherapy. Detailed study-level information on anticancer treatments is provided in Supplemental Table 4.2. Study-level characteristics are presented to contextualize clinical heterogeneity before quantitative synthesis.

### Risk of bias

In the assessment of study quality using the ROBINS-I tool, all included studies were judged at moderate risk of bias (Supplemental Table 5). This was largely due to their observational design, although it is important to note that nearly all of them employed propensity score matching or other adjustment strategies to minimize confounding.

### Key primary endpoints

**Figure 1:**
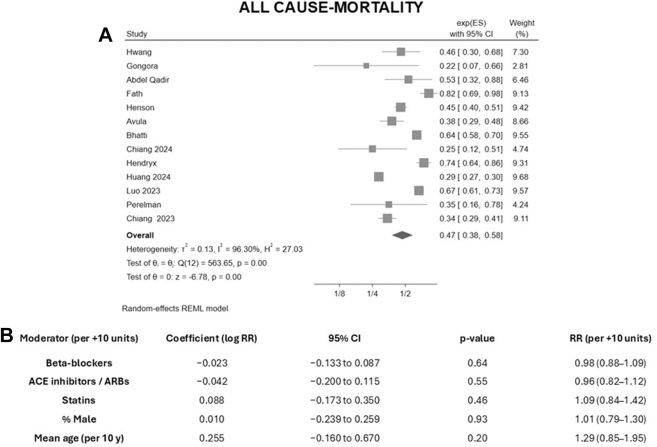
All-Cause Mortality and Study-Level Meta-Regression Analyses (A) Random-effects meta-analysis (REML model) of SGLT2 inhibitors vs controls for all-cause mortality. (B) Univariable meta-regressions testing beta-blocker, ACEI/ARB, statin use, sex, and mean age as study-level moderators (effect per 10% or 10-year increase). ACEI/ARB = angiotensin-converting enzyme inhibitor or angiotensin receptor blocker; exp(ES) = exponentiated effect size; REML = restricted maximum likelihood; RR = risk ratio.

**Figure 2:**
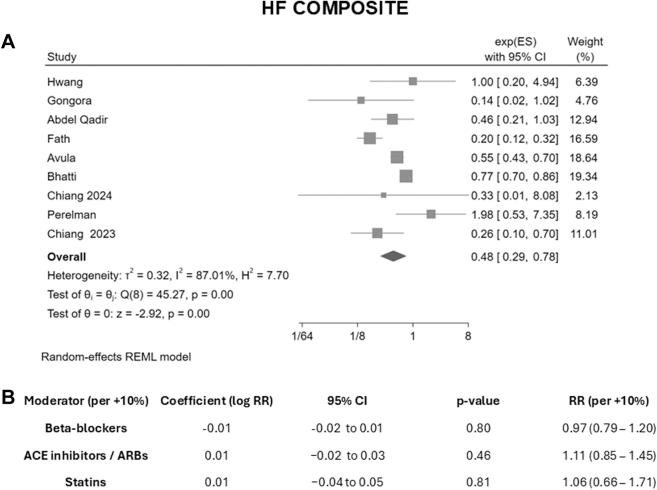
Heart Failure Composite and Study-Level Meta-Regression Analyses (A) Random-effects meta-analysis (REML model) of SGLT2 inhibitors vs controls for the composite of incident HF and/or HF hospitalization. (B) Univariable meta-regressions testing beta-blocker, ACEI/ARB, and statin use as study-level moderators (effect per 10% increase). HF = heart failure; other abbreviations as in Figure 1.

All-cause mortality was reduced in the SGLT2i arm (random-effects REML; RR: 0.47; 95% CI: 0.38-0.58; *P* < 0.001) with substantial heterogeneity (τ^2^ ≈ 0.13; I^2^ = 96.30%; *P* < 0.001) (Figure 1A). Visual inspection of the funnel plot revealed no evident asymmetry, and Egger test was nonsignificant (*P* ≈ 0.12) (Supplemental Figure 1.1). Meta-regressions did not identify meaningful effect modification of the mortality association by background cardioprotective therapy, age, or sex (all *P* values for interactions > 0.05) (Figure 1B). For the HF composite (incident HF and/or HF hospitalization), SGLT2i were associated with a lower risk (RR: 0.48; 95% CI: 0.29-0.78; *P* < 0.001), with substantial heterogeneity (τ^2^ = 0.32; I^2^ = 87.01%; *P* < 0.001) (Figure 2A). The funnel plot appeared broadly symmetric, and Egger test did not indicate small-study effects (*P* ≈ 0.86) (Supplemental Figure 1.2). Consistently, none of the study-level prevalences of beta-blocker, ACEI/ARB, or statin use significantly modified the association between SGLT2i therapy and HF outcomes (all *P* > 0.05) (Figure 2B). Consistent with the substantial between-study heterogeneity, Doi plots and LFK indices showed major asymmetry for both all-cause mortality and the HF composite (Supplemental Figures 1.1 and 1.2). Furthermore, 95% prediction intervals were calculated. The prediction interval for all-cause mortality ranged from 0.21 to 1.05, whereas that for the HF composite ranged from 0.07 to 2.28.

### Secondary and safety outcomes

**Figure 3:**
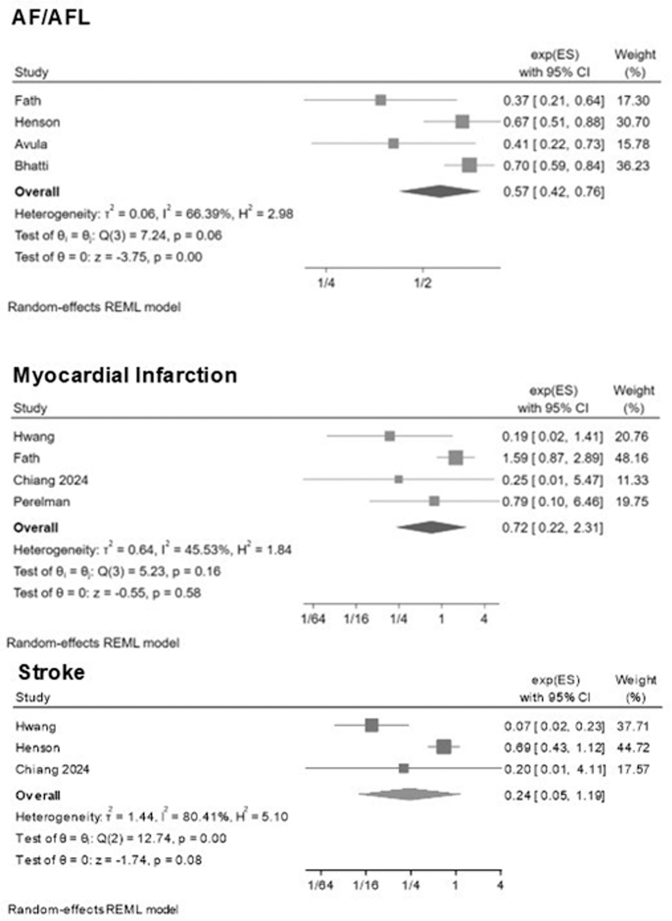
**Forest Plots for Atrial Fibrillation/Atrial Flutter, Myocardial Infarction, and Stroke** AF/AFL = atrial fibrillation/atrial flutter; other abbreviation as in Figure 1.

For the composite of atrial fibrillation/atrial flutter, SGLT2i were associated with a significantly lower risk compared with controls (RR: 0.57; 95% CI: 0.42-0.76; *P* < 0.001), and moderate-to-high heterogeneity was observed (I^2^ = 66.4%) (Figure 3). Visual inspection of the funnel plot suggested some degree of asymmetry (Supplemental Figure 1.3). Egger test indicated potential small-study effects (*P* = 0.009).

**Figure 4:**
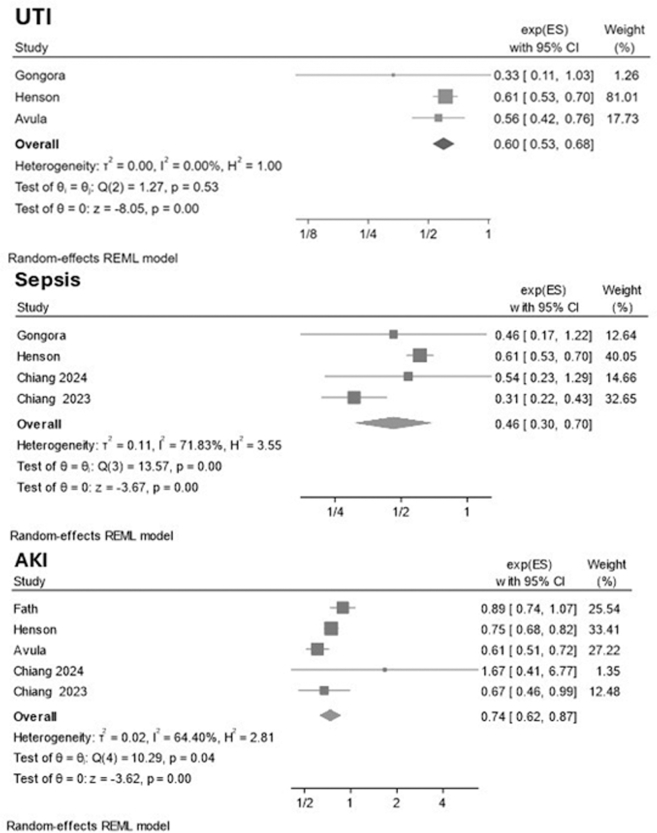
**Random-Effects Meta-Analysis of the Association Between SGLT2 Inhibitors and AKI, UTI, and Sepsis** Effect sizes are reported as exponentiated effect estimates (exp[ES]) with 95% CI. AKI = acute kidney injury; UTI = urinary tract infection; other abbreviation as in Figure 1.

For myocardial infarction, SGLT2i were not associated with a significant difference in risk compared with controls (RR: 0.72; 95% CI: 0.22-2.31; *P* = 0.58) (Figure 3). Heterogeneity was moderate (τ^2^ = 0.64; I^2^ = 45.5%; *P* = 0.16). The funnel plot suggested possible small-study effects, with Egger test reaching nominal significance (*P* = 0.043) (Supplemental Figure 1.4). Similar results were observed for stroke: SGLT2i were not associated with a significant reduction in risk compared with controls (RR: 0.24; 95% CI: 0.05-1.19; *P* = 0.08) (Figure 3). Heterogeneity was substantial (τ^2^ = 1.44; I^2^ = 80.4%; *P* = 0.002). The funnel plot appeared symmetric, and Egger test did not show a small-study effect (*P* = 0.67) (Supplemental Figure 1.5). Safety outcomes showed no signal of harm with SGLT2i. For UTIs, SGLT2i were associated with a reduced risk compared with controls (RR: 0.60; 95% CI: 0.53-0.68; *P* < 0.001), with no heterogeneity detected (τ^2^ = 0.00; I^2^ = 0%) (Figure 4). The funnel plot appeared symmetric, and Egger test indicated no evidence of small-study effects (*P* = 0.26) (Supplemental Figure 1.6). For sepsis, SGLT2i were not associated with an increased risk compared with controls (RR: 0.46; 95% CI: 0.30-0.70; *P* < 0.001), with moderate heterogeneity (τ^2^ = 0.11; I^2^ = 71.8%) (Figure 4). The funnel plot appeared symmetric, and Egger test confirmed the absence of small-study effects (*P* = 0.93) (Supplemental Figure 1.7). AKI was reduced with SGLT2i compared with controls (RR: 0.74; 95% CI: 0.62-0.87; *P* < 0.001), with moderate heterogeneity (τ^2^ = 0.02; I^2^ = 64.4%) (Figure 4). The funnel plot was symmetric, and Egger test did not indicate small-study effects (*P* = 0.39) (Supplemental Figure 1.8).

### Sensitivity analysis

In cohorts where all patients received at least 1 cycle of anthracycline-based chemotherapy, SGLT2i remained significantly associated with reduced all-cause mortality (RR: 0.52; 95% CI: 0.38-0.72; *P* < 0.001; I^2^ = 83.6%) (Supplemental Figure 2.1) and lower risk of the HF composite (RR: 0.22; 95% CI: 0.11-0.43; *P* < 0.001; I^2^ = 47%) (Supplemental Figure 2.2). Furthermore, as shown in Supplemental Figures 2.3 and 2.4, univariable meta-regression analyses confirmed the robustness of the treatment effect of SGLT2i across key study-level covariates. For all-cause mortality and the HF composite, no significant effect modification was observed according to the prevalence of dyslipidemia, hypertension, breast, gastrointestinal, or hematologic cancer, or follow-up duration. Finally, pooling adjusted HRs for all-cause mortality confirmed a significant association between SGLT2i use and reduced mortality (random-effects HR: 0.46; 95% CI: 0.35-0.62; *P* < 0.001), despite substantial between-study heterogeneity (I^2^ = 96.61%) (Supplemental Figure 2.5). The 95% prediction interval was wide (0.31-1.08), indicating considerable variability in the direction and magnitude of the association across studies. A detailed overview of covariates included in each adjusted model is provided in Supplemental Table 4.2. To minimize potential bias arising from overlapping source populations, particularly from the TriNetX registry and studies from the Chiang group, we performed a sensitivity analysis retaining only 1 representative study per data source, prioritizing those with the largest sample size. For all-cause mortality, the association remained significant (RR: 0.47; 95% CI: 0.36-0.61; *P* < 0.001), with persistent substantial heterogeneity (I^2^ = 96.8%) (Supplemental Figure 2.6). Similarly, for the HF composite, the association remained statistically significant (RR: 0.42; 95% CI: 0.19-0.93; *P* = 0.03), although heterogeneity remained high (I^2^ = 80.5%) (Supplemental Figure 2.7). Overall, these findings were consistent with the main analysis, suggesting that the observed associations might not be driven by potential overlap across data sources. Our last sensitivity analysis was restricted to HF hospitalization-only events and yielded consistent results with the main analysis (RR: 0.55; 95% CI: 0.40-0.77; I^2^ = 70.4%; *P* < 0.001) (Supplemental Figure 2.8).

## Discussion

### Main findings

Our meta-analysis reveals 3 key findings, summarized in the Central Illustration. First, in adults with cancer, SGLT2i were associated with lower all-cause mortality and fewer HF events (incident HF and/or HF hospitalization). Second, the mortality and HF benefits appeared independent of background cardioprotective therapy—beta-blockers, ACEI/ARBs, and statins—and, for mortality, were not modified by age or sex. Third, across the safety endpoints that were most reported by the included studies, SGLT2i were not linked to excess risk; rather, pooled estimates suggested lower rates of UTI, sepsis, and AKI, whereas myocardial infarction and stroke were overall neutral. Our results align closely with those of recent meta-analyses. Novo et al,^20^ in 104,327 patients with diabetes and cancer, reported significant reductions in all-cause mortality and HF hospitalization among SGLT2i users (RR: 0.47 and 0.44, respectively). Similarly, Bhalraam et al.^21^ found lower risk of new HF (RR: 0.29) and HF hospitalizatxion (RR: 0.49) with SGLT2i treatment in 88,273 patients with cancer. Results from our work are in line with these findings, adding, however, 2 new relevant findings: 1) to the best of our knowledge, the present meta-analysis represents the largest sample to date (107,126 patients), exceeding prior totals; and 2) study-level meta-regression suggests that the observed mortality and HF benefits might not be influenced by concomitant use of beta-blockers, ACEI/ARBs, or statins and, for mortality, also appear independent of age and sex.

**Central Illustration:**
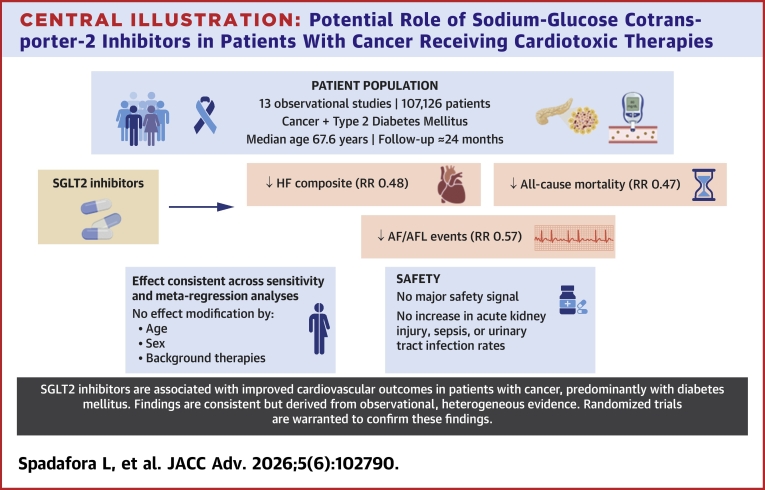
**Potential Role of Sodium-Glucose Cotransporter-2 Inhibitors in Patients With Cancer Receiving Cardiotoxic Therapies** Across observational studies, SGLT2i use was associated with reduced all-cause mortality and improved heart failure (HF) outcomes, with consistent findings across sensitivity analyses. These associations appeared independent of age, sex, and background cardioprotective therapies. Overall, the evidence remains observational and heterogeneous, and results should be considered hypothesis-generating. Ongoing randomized clinical trials are needed to clarify the role of SGLT2i as a cardioprotective strategy in cardio-oncology. AF/AFL = atrial fibrillation/atrial flutter; RR = risk ratio.

### Prior literature and ongoing research

For the prevention of chemotherapy-related cardiotoxicity, current guidelines recommend several therapeutic strategies, including beta-blockers, statins, ACEI/ARBs, dexrazoxane, liposomal anthracyclines, and chemotherapy dose reduction.^4^^,^^5^^,^^23^ Moreover, current guidelines emphasize the need to identify new cardioprotective strategies for patients at high cardiovascular risk who are candidates for chemotherapy.^23^ As a result, there is active work across the scientific community to develop both pharmacologic and nonpharmacologic approaches, although concrete advances have been slow to emerge.^25^ For instance, trials on neurohormonal modulation with sacubitril/valsartan for chemotherapy-related cardiotoxicity have produced mixed results,^26^^,^^27^ The biological rationale for SGLT2i in cardio-oncology is increasingly compelling.^3^ Beyond glycemic effects, SGLT2i modulate myocardial energetics and ion homeostasis, promote autophagy and mitophagy, dampen inflammatory and NLRP3-inflammasome signaling, and mitigate oxidative stress—mechanisms repeatedly implicated in anthracycline and HER2-therapy cardiotoxicity.^28^^,^^29^ Intriguingly, a growing body of preclinical research proposes that SGLT2 inhibition may also interact with cancer biology (eg, AMP-activated protein kinase [AMPK] and Hippo/Yes-associated protein [Hippo/YAP] pathways), although these anticancer effects remain exploratory and far from clinically established.^3^^,^^28^^,^^30^ Emerging and ongoing clinical trials will be decisive. EMPACT (NCT05271162) is a randomized, multicenter, double-blind study testing whether prophylactic empagliflozin prevents LVEF decline in patients slated to receive high cumulative anthracycline doses; it will enroll 220 participants with preserved ejection fraction and no prior HF, with serial echocardiography and cardiac magnetic resonance to quantify left ventricular function, and a composite of clinical events as secondary outcomes.^31^ HER2HEART-US (NCT06844669) is a pilot, 2×2 factorial trial in patients beginning HER2-directed therapy, designed to assess feasibility and preliminary efficacy and safety of carvedilol and/or empagliflozin vs usual care—probing whether a pragmatic cardioprotective “bundle” is workable in routine oncology. PROTECT (NCT06341842), a multicenter phase II randomized controlled trial in early-stage breast cancer, will evaluate whether dapagliflozin can reduce anthracycline- and/or trastuzumab-associated cardiotoxicity while also characterizing systemic inflammatory and metabolic effects in this setting.^32^ Other observations from our meta-analysis deserve attention. The associations between SGLT2i and both all-cause mortality and the HF composite endpoints were maintained irrespective of sex and age. Indeed, as suggested by several studies, sex-related differences may exist in the development of cardiotoxicity—differences that SGLT2i could help mitigate.^33^, ^34^, ^35^ The reported safety endpoints did not suggest an increased risk with SGLT2i; pooled estimates were directionally favorable for UTI, sepsis, and AKI. However, these findings should be interpreted with caution. Infection-related outcomes were reported in a limited number of studies and were often based on administrative data, with potential for surveillance bias and residual confounding.

### Study limitations

Several limitations should be considered when interpreting these findings. For the primary analyses, although pooled estimates were statistically significant, between-study heterogeneity was substantial and prediction intervals were wide. In addition, the median follow-up duration of approximately 24 months may not be sufficient to fully capture long-term effects. Meta-regression was conducted at the study level and is therefore subject to ecological bias. The lack of individual-patient data precluded adjustment for important clinical variables, including cancer stage, radiation exposure, cumulative anthracycline dose, and use of cardioprotective strategies such as dexrazoxane or liposomal formulations. Accordingly, meta-regression findings should be interpreted as exploratory. Although several study-level covariates were examined, they did not fully explain the observed heterogeneity. Baseline characteristics varied substantially across studies, particularly with respect to cardiometabolic comorbidities, suggesting that differences in patient profiles may have contributed to between-study variability. Residual confounding cannot be excluded, as unmeasured or incompletely captured factors—such as treatment adherence, access to care, and variations in clinical management—may have influenced the observed associations. Concomitant use of cardioprotective therapies was common, and potential synergistic or confounding effects with SGLT2i cannot be excluded, despite meta-regression analyses. For some endpoints, the limited number of contributing studies reduced statistical power and constrained the interpretability of small-study effect analyses. Although Doi plots and LFK indices suggested asymmetry for all-cause mortality and the HF composite, this was not consistently supported by funnel plot inspection or Egger tests. Definitions of the HF composite varied across studies and were based on administrative or clinical criteria, potentially contributing to heterogeneity in endpoint ascertainment. To address this, we performed a sensitivity analysis restricted to HF hospitalization-only events, which yielded consistent results. Data on HF outcomes were not stratified according to HF phenotype (heart failure with preserved ejection fraction [HFpEF], heart failure with mildly reduced ejection fraction [HFmrEF], and heart failure with reduced ejection fraction [HFrEF]), limiting the ability to assess whether the observed associations differ across clinically distinct HF subtypes. The possibility of overlapping populations should also be considered, particularly among studies based on the TriNetX platform; however, sensitivity analyses accounting for this issue did not materially alter the findings. In addition, information on the specific type of SGLT2i was inconsistently reported across studies, precluding any analysis of potential drug-specific effects. Similarly, the timing of SGLT2i initiation in relation to cancer therapy (before, during, or after treatment) was heterogeneous and not consistently available at the patient level, limiting the ability to distinguish preventive from therapeutic effects. Information on race and ethnicity was inconsistently reported, limiting assessment of differential effects across populations. This is particularly relevant given known disparities in cardiovascular outcomes, highlighting the need for more inclusive and representative data in cardio-oncology research.^36^, ^37^, ^38^, ^39^ Finally, although variability across studies was anticipated and addressed using random-effects models, this approach captures between-study variability but does not fully resolve challenges related to clinical heterogeneity. Accordingly, the observed associations should be interpreted as average effects across heterogeneous populations rather than as directly applicable to specific clinical contexts.

### Interpretation and clinical implications

From a clinical perspective, these findings suggest that SGLT2i may provide a cardioprotective effect that appears independent of background therapies commonly used in cardio-oncology, particularly for HF-related outcomes. In addition, SGLT2i appear associated with mortality benefits. The available evidence is observational, heterogeneous, and subject to residual confounding; therefore, the findings should be considered hypothesis-generating rather than definitive. In this context, the meta-analysis represents a conditional, secondary layer of synthesis, summarizing associations across diverse clinical scenarios rather than defining a single, generalizable treatment effect. Despite these many limitations, the consistency of the observed associations, together with the established role of SGLT2i in patients at elevated cardiovascular risk, suggests that their use in patients with diabetes and cancer may be reasonable in this setting. These findings may therefore support a more proactive consideration of SGLT2i in cardio-oncology, while awaiting confirmation from randomized clinical trials.

## Conclusions

This systematic review and meta-analysis of observational studies suggests that, in patients with cancer, SGLT2i use appears to be associated with lower all-cause mortality and reduced HF outcomes. These associations appeared consistent across multiple sensitivity and meta-regression analyses. However, given the observational nature of the available evidence and the substantial heterogeneity, these findings should be interpreted as hypothesis-generating. Ongoing randomized clinical trials are needed to determine whether SGLT2i may represent an effective cardioprotective strategy in cardio-oncology.

## Funding support and author disclosures

This work was partially supported by a grant from 10.13039/501100004271Sapienza University of Rome (RD12318AAABE3A08). Dr Sciarretta received honoraria for speeches and/or advisory board participation from Novo Nordisk and AstraZeneca. Dr Biondi Zoccai has consulted, lectured, and/or served as an advisory board member for Abiomed, Advanced Nanotherapies, Amarin, AstraZeneca, Balmed, Cardionovum, Cepton, Crannmedical, Endocore Lab, Eukon, Guidotti, Innovheart, Menarini, Microport, Opsens Medical, Servier, Synthesa, Terumo, and Translumina, outside the present work. Dr Abbate has served as a paid consultant to Kiniksa, Monte Rosa, and Novo Nordisk. All other authors have reported that they have no relationships relevant to the contents of this paper to disclose.

## References

[1] McDonagh T.A., Metra M., Adamo M. (2021). 2021 ESC Guidelines for the diagnosis and treatment of acute and chronic heart failure. Eur Heart J.

[2] Aristizabal-Colorado D., Ocampo-Posada M., Rivera-Martinez W.A. (2024). SGLT2 inhibitors and how they work beyond the glucosuric effect. State of the art. Am J Cardiovasc Drugs.

[3] Camilli M., Viscovo M., Maggio L. (2025). Sodium-glucose cotransporter 2 inhibitors and the cancer patient: from diabetes to cardioprotection and beyond. Basic Res Cardiol.

[4] Camilli M., Cipolla C.M., Dent S., Minotti G., Cardinale D.M. (2024). Anthracycline cardiotoxicity in adult cancer patients: JACC: cardiooncology state-of-the-art review. JACC CardioOncol.

[5] Spadafora L., Di Muro F.M., Intonti C. (2025). Lifestyle and pharmacological interventions to prevent anthracycline-related cardiotoxicity in cancer patients. J Cardiovasc Dev Dis.

[6] Cardinale D., Colombo A., Bacchiani G. (2015). Early detection of anthracycline cardiotoxicity and improvement with heart failure therapy. Circulation.

[7] Gongora C.A., Drobni Z.D., Quinaglia Araujo Costa Silva T. (2022). Sodium-glucose Co-Transporter-2 inhibitors and cardiac outcomes among patients treated with anthracyclines. JACC Heart Fail.

[8] Daniele A.J., Gregorietti V., Costa D., Lopez-Fernandez T. (2024). Use of EMPAgliflozin in the prevention of CARDiotoxicity: the EMPACARD - PILOT trial. Cardiooncology.

[9] Abdel-Qadir H., Carrasco R., Austin P.C. (2023). The Association of Sodium-glucose cotransporter 2 inhibitors with cardiovascular outcomes in anthracycline-treated patients with cancer. JACC CardioOncol.

[10] Avula V., Sharma G., Kosiborod M.N. (2024). SGLT2 inhibitor use and risk of clinical events in patients with cancer therapy-related cardiac dysfunction. JACC Heart Fail.

[11] Bhatti A.W., Patel R., Dani S.S. (2024). SGLT2i and primary prevention of cancer therapy-related cardiac dysfunction in patients with diabetes. JACC CardioOncol.

[12] Chiang C.H., Chiang C.H., Hsia Y.P. (2024). The impact of sodium-glucose cotransporter-2 inhibitors on outcome of patients with diabetes mellitus and colorectal cancer. J Gastroenterol Hepatol.

[13] Fath A.R., Aglan M., Aglan A. (2024). Cardioprotective potential of sodium-glucose Cotransporter-2 inhibitors in patients with cancer treated with anthracyclines: an observational study. Am J Cardiol.

[14] Hendryx M., Dong Y., Ndeke J.M., Luo J. (2022). Sodium-Glucose Cotransporter 2 (SGLT2) inhibitor initiation and hepatocellular carcinoma prognosis. PLoS One.

[15] Huang Y.M., Chen W.M., Jao A.T., Chen M., Shia B.C., Wu S.Y. (2024). Effects of SGLT2 inhibitors on clinical cancer survival in patients with type 2 diabetes. Diabetes Metab.

[16] Hwang H.J., Kim M., Jun J.E., Yon D.K. (2023). Sodium-glucose cotransporter-2 inhibitors improve clinical outcomes in patients with type 2 diabetes mellitus undergoing anthracycline-containing chemotherapy: an emulated target trial using nationwide cohort data in South Korea. Sci Rep.

[17] Kuo H.H., Wang K.T., Chen H.H. (2024). Cardiovascular outcomes associated with SGLT2 inhibitor therapy in patients with type 2 diabetes mellitus and cancer: a systematic review and meta-analysis. Diabetol Metab Syndr.

[18] Luo J., Hendryx M., Dong Y. (2023). Sodium-Glucose Cotransporter 2 (SGLT2) inhibitors and non-small cell lung cancer survival. Br J Cancer.

[19] Perelman M.G., Brzezinski R.Y., Waissengrin B. (2024). Sodium-glucose co-transporter-2 inhibitors in patients treated with immune checkpoint inhibitors. Cardiooncology.

[20] Novo G., Madaudo C., Cannata A. (2025). Effects of sodium-glucose cotransporter 2 inhibitors in patients with cancer and diabetes mellitus: a systematic review and meta-analysis. Eur Heart J Cardiovasc Pharmacother.

[21] Bhalraam U., Veerni R.B., Paddock S. (2025). Impact of sodium-glucose cotransporter-2 inhibitors on heart failure outcomes in cancer patients and survivors: a systematic review and meta-analysis. Eur J Prev Cardiol.

[22] Reshadmanesh T., Mohebi R., Behnoush A.H. (2025). The effects of sodium-glucose cotransporter-2 inhibitors in chemotherapy-induced cardiotoxicity and mortality in patients with cancer: a systematic review and meta-analysis. Cardiooncology.

[23] Lyon A.R., Lopez-Fernandez T., Couch L.S. (2022). 2022 ESC Guidelines on cardio-oncology developed in collaboration with the European Hematology Association (EHA), the European Society for Therapeutic Radiology and Oncology (ESTRO) and the International Cardio-Oncology Society (IC-OS). Eur Heart J.

[24] Abbas M.T., Farina J.M., Razaghi M. (2025). The association between sodium-glucose cotransporter 2 inhibitors treatment and survival in patients with cancer therapy-related cardiac dysfunction. Eur J Prev Cardiol.

[25] Fabiani I., Chianca M., Aimo A. (2024). Use of new and emerging cancer drugs: what the cardiologist needs to know. Eur Heart J.

[26] Omland T., Heck S.L., Holte E. (2025). Sacubitril-Valsartan and prevention of cardiac dysfunction during adjuvant breast cancer therapy: the PRADA II randomized clinical trial. Circulation.

[27] Mecinaj A., Vinje-Jakobsen V., Ngo D.T.M., Sverdlov A.L., Myhre P.L. (2025). The SARAH trial: more evidence on the role of neurohormonal blockers in prevention of anthracycline-induced cardiotoxicity. Heart Fail Rev.

[28] Dabour M.S., George M.Y., Daniel M.R., Blaes A.H., Zordoky B.N. (2024). The cardioprotective and anticancer effects of SGLT2 inhibitors: JACC: cardiooncology state-of-the-art review. JACC CardioOncol.

[29] Quagliariello V., De Laurentiis M., Rea D. (2021). The SGLT-2 inhibitor empagliflozin improves myocardial strain, reduces cardiac fibrosis and pro-inflammatory cytokines in non-diabetic mice treated with doxorubicin. Cardiovasc Diabetol.

[30] Karakasis P., Patoulias D., Fragakis N. (2025). Balancing promise and evidence: critical perspectives on SGLT2 inhibitors for cardiotoxicity prevention. JACC CardioOncol.

[31] Osataphan N., Abdel-Qadir H., Zebrowska A.M., Borowiec A. (2024). Sodium-glucose cotransporter 2 inhibitors during cancer therapy: benefits, risks, and ongoing clinical trials. Curr Oncol Rep.

[32] Greco A., Quagliariello V., Rizzo G. (2025). SGLT2i Dapagliflozin in primary prevention of chemotherapy induced cardiotoxicity in breast cancer patients treated with neo-adjuvant anthracycline-based chemotherapy +/- trastuzumab: rationale and design of the multicenter PROTECT trial. Cardiooncology.

[33] Cadeddu Dessalvi C., Pepe A., Penna C. (2019). Sex differences in anthracycline-induced cardiotoxicity: the benefits of estrogens. Heart Fail Rev.

[34] Kheradkhah G., Sheibani M., Kianfar T., Toreyhi Z., Azizi Y. (2024). A comprehensive review on the effects of sex hormones on chemotherapy-induced cardiotoxicity: are they lucrative or unprofitable?. Cardiooncology.

[35] Meiners B., Shenoy C., Zordoky B.N. (2018). Clinical and preclinical evidence of sex-related differences in anthracycline-induced cardiotoxicity. Biol Sex Differ.

[36] Franciosa J.A., Ferdinand K.C., Yancy C.W., Consensus Statement on Heart Failure in African Americans Writing Group (2010). Treatment of heart failure in African Americans: a consensus statement. Congest Heart Fail.

[37] Breathett K., Liu W.G., Allen L.A. (2018). African Americans are less likely to receive care by a cardiologist during an intensive care unit admission for heart failure. JACC Heart Fail.

[38] Osei Baah F., Sharda S., Davidow K. (2024). Social determinants of health in cardio-oncology: multi-level strategies to overcome disparities in care: JACC: cardiooncology state-of-the-art review. JACC CardioOncol.

[39] Carnethon M.R., Pu J., Howard G. (2017). Cardiovascular health in African Americans: a scientific statement from the American Heart Association. Circulation.

