# Supplementary material for: SGLT2 Inhibitors in Cardio-Oncology: A Systematic Review and Meta-Analysis
Source: JACC Adv. 2026 May 14;5(6):102790. doi: 10.1016/j.jacadv.2026.102790 (PMC13202275; doi:10.1016/j.jacadv.2026.102790)
Supplement: Supplementary material [file mmc1.docx]

**SUPPLEMENTARY MATERIAL**

**SGLT2 inhibitors in cardio-oncology: a systematic review and meta-analysis.**

**Luigi Spadafora et al.**

**Table of Contents**

**LIST OF INCLUDED STUDIES**

**Supplementary Table 1. Full electronic search strategy through August 2025**

**Supplementary Table 2. Endpoints Availability and Definitions Across Trials**

**Supplementary Table 3. PRISMA flowchart**

**Supplementary Table 4. Baseline features of the included studies**

**Supplementary Table 5. Risk-of-bias assessment**

**Supplementary Figures 1. Funnel plots**

**Supplementary Figures 2. Sensitivity analyses**

**LIST OF INCLUDED STUDIES**

1. **ABDEL-QADIR et al. (2023)**

Abdel-Qadir H, Carrasco R, Austin PC, Chen Y, Zhou L, Fang J, Su HMH, Lega IC, Kaul P, Neilan TG, Thavendiranathan P. The Association of Sodium-Glucose Cotransporter 2 Inhibitors With Cardiovascular Outcomes in Anthracycline-Treated Patients With Cancer. JACC Cardio-Oncology 2023 May 2;5(3):318-328. doi: 10.1016/j.jaccao.2023.03.011. PMID: 37397088; PMCID: PMC10308059.

1. **AVULA et al. (2024)**

Avula V, Sharma G, Kosiborod MN, Vaduganathan M, Neilan TG, Lopez T, Dent S, Baldassarre L, Scherrer-Crosbie M, Barac A, Liu J, Deswal A, Khadke S, Yang EH, Ky B, Lenihan D, Nohria A, Dani SS, Ganatra S. SGLT2 Inhibitor Use and Risk of Clinical Events in Patients With Cancer Therapy-Related Cardiac Dysfunction. JACC Heart Fail. 2024 Jan;12(1):67-78. doi: 10.1016/j.jchf.2023.08.026. Epub 2023 Oct 25. PMID: 37897456.

1. **BHATTI et al. (2024)**

Bhatti AW, Patel R, Dani SS, Khadke S, Makwana B, Lessey C, Shah J, Al-Husami Z, Yang EH, Thavendiranathan P, Neilan TG, Sadler D, Cheng RK, Dent SF, Liu J, Lopez-Fernandez T, Herrmann J, Scherrer-Crosbie M, Lenihan DJ, Hayek SS, Ky B, Deswal A, Barac A, Nohria A, Ganatra S. SGLT2i and Primary Prevention of Cancer Therapy-Related Cardiac Dysfunction in Patients With Diabetes. JACC Cardio-Oncol. 2024 Sep 22;6(6):863-875. doi: 10.1016/j.jaccao.2024.08.001. PMID: 39801650; PMCID: PMC11711834.

1. **CHIANG et al. (2023)**

Chiang CH, Chiang CH, Chiang CH, Ma KS, Peng CY, Hsia YP, Horng CS, Chen CY, Chang YC, See XY, Chen YJ, Wang SS, Suero-Abreu GA, Peterson LR, Thavendiranathan P, Armand P, Peng CM, Shiah HS, Neilan TG. Impact of sodium-glucose cotransporter-2 inhibitors on heart failure and mortality in patients with cancer. Heart. 2023 Feb 23;109(6):470-477. doi: 10.1136/heartjnl-2022-321545. PMID: 36351793; PMCID: PMC10037540.

1. [**CHIANG et al.**](https://pubmed.ncbi.nlm.nih.gov/?term=STEP-HFpEF+Trial+Committees+and+Investigators%5BCorporate+Author%5D)**(2024)**

Chiang CH, Chiang CH, Hsia YP, Jaroenlapnopparat A, Horng CS, Wong KY, Wang SS, Chang YC, Chen BS, Luan YZ, Wang CH, Neilan TG, Chiang CH, Peng CM, Shiah HS. The impact of sodium-glucose cotransporter-2 inhibitors on outcome of patients with diabetes mellitus and colorectal cancer. J Gastroenterol Hepatol. 2024 May;39(5):902-907. doi: 10.1111/jgh.16498. Epub 2024 Jan 31. PMID: 38296226.

1. **FATH et al. (2024)**

Fath AR, Aglan M, Aglan A, Chilton RJ, Trakhtenbroit A, Al-Shammary OA, Oppong-Nkrumah O, Lenihan DJ, Dent SF, Otchere P. Cardioprotective Potential of Sodium-Glucose Cotransporter-2 Inhibitors in Patients With Cancer Treated With Anthracyclines: An Observational Study. Am J Cardiol. 2024 Jul 1;222:175-182. doi: 10.1016/j.amjcard.2024.04.032. Epub 2024 Apr 30. PMID: 38692401.

1. **GONGORA et al. (2022)**

Gongora CA, Drobni ZD, Quinaglia Araujo Costa Silva T, Zafar A, Gong J, Zlotoff DA, Gilman HK, Hartmann SE, Sama S, Nikolaidou S, Suero-Abreu GA, Jacobsen E, Abramson JS, Hochberg E, Barnes J, Armand P, Thavendiranathan P, Nohria A, Neilan TG. Sodium-Glucose Co-Transporter-2 Inhibitors and Cardiac Outcomes Among Patients Treated With Anthracyclines. JACC Heart Fail. 2022 Aug;10(8):559-567. doi: 10.1016/j.jchf.2022.03.006. Epub 2022 Jun 8. PMID: 35902159; PMCID: PMC9638993.

1. **HENDRYX et al. (2022)**

Hendryx M, Dong Y, Ndeke JM, Luo J. Sodium-glucose cotransporter 2 (SGLT2) inhibitor initiation and hepatocellular carcinoma prognosis. PLoS One. 2022 Sep 12;17(9):e0274519. doi: 10.1371/journal.pone.0274519. PMID: 36094949; PMCID: PMC9467321.

1. **HENSON et al. (2024)**

Henson, Benjamin D. BS*; Bale-Neary, Claudia A. BA*; Mecaskey, Ryan BS*; Gbujie, Ogechi BA*; Zhan, Michelle BA*; Rao, Krishnasree MD†,‡; Carbone, Salvatore PhD, RD, FHFSA, FASPEN‡,§. Sodium–Glucose Cotransporter 2 Inhibitors, Malnutrition, Cachexia, and Survival in Patients With Heart Failure With a History of Anthracycline Treatment. Journal of Cardiovascular Pharmacology 84(5):p 486-489, November 2024. | DOI: 10.1097/FJC.0000000000001620.

1. **HUANG et al. (2024)**

Huang YM, Chen WM, Jao AT, Chen M, Shia BC, Wu SY. Effects of SGLT2 inhibitors on clinical cancer survival in patients with type 2 diabetes. Diabetes Metab. 2024 Jan;50(1):101500. doi: 10.1016/j.diabet.2023.101500. Epub 2023 Nov 28. PMID: 38036054.

1. **HWANG et al. (2023)**

Hwang, HJ., Kim, M., Jun, J.E. *et al.* Sodium-glucose cotransporter-2 inhibitors improve clinical outcomes in patients with type 2 diabetes mellitus undergoing anthracycline-containing chemotherapy: an emulated target trial using nationwide cohort data in South Korea. *Sci Rep* 13, 21756 (2023).

1. **LUO et al. (2023)**

Luo J, Hendryx M, Dong Y. Sodium-glucose cotransporter 2 (SGLT2) inhibitors and non-small cell lung cancer survival. Br J Cancer. 2023 Apr;128(8):1541-1547. doi: 10.1038/s41416-023-02177-2. Epub 2023 Feb 10. PMID: 36765176; PMCID: PMC10070339.

1. **PERELMAN et al. (2024)**

Perelman MG, Brzezinski RY, Waissengrin B, Leshem Y, Bainhoren O, Rubinstein TA, Perelman M, Rozenbaum Z, Havakuk O, Topilsky Y, Banai S, Wolf I, Laufer-Perl M. Sodium-glucose co-transporter-2 inhibitors in patients treated with immune checkpoint inhibitors. Cardio-Oncology. 2024 Jan 11;10(1):2. doi: 10.1186/s40959-023-00199-6. PMID: 38212825; PMCID: PMC10782769.**Supplementary Table 1. Full electronic search strategy through August 2025.**

| **Research** | **Query** | **Items found** |
| --- | --- | --- |
| 1 | ("Sodium-Glucose Transporter 2 Inhibitors"[Mesh] OR sglt2[tiab] OR empagliflozin[tiab] OR dapagliflozin[tiab]) AND (cardio-oncology[tiab] OR cardiotoxic*[tiab] OR Neoplasms[Mesh]) | 365 |
| 2 | (("Sodium-Glucose Transporter 2 Inhibitors"[Mesh] OR "SGLT2 inhibitor*"[tiab] OR empagliflozin[tiab] OR dapagliflozin[tiab] OR canagliflozin[tiab]) AND (Neoplasms[Mesh] OR cancer*[tiab] OR anthracycline*[tiab] OR trastuzumab[tiab] OR "immune checkpoint inhibitor*"[tiab]) AND (cardiotoxic*[tiab] OR "Heart Failure"[Mesh] OR MACE[tiab] OR mortality[tiab])) NOT (animals[mh] NOT humans[mh]) | 125 |

**Supplementary Table 2. Endpoints Availability and Definitions Across Studies.**

|  | **All-Cause Mortality** | **HF Composite** | **MI** | **AKI** | **AF/AFL** | **Stroke** | **Sepsis** | **UTI** |
| --- | --- | --- | --- | --- | --- | --- | --- | --- |
| **Abdel Qadir** | • | New onset-HF and/or HF hospitalization | NA | NA | NA | NA | NA | NA |
| **Avula** | • | Defined as HF hospitalization/exacerbation (ICD-10 HF code or need for IV loop diuretics). | NA | AKI by ICD-10 codes | A composite of AF and AFL | NA | NA | UTI by ICD-10 codes |
| **Bhatti** | • | HF exacerbation/hospitalization | NA | NA | Reported as new-onset AF or AFL | NA | NA | NA |
| **Chiang 2023** | • | Hospitalization for HF | NA | AKI | NA | NA | • | NA |
| **Chiang 2024** | • | New-onset HF | Myocardial infarction was defined according to administrative (ICD-based) definitions. | AKI captured as a serious adverse event (ICD/EHR coding) | NA | • |  | NA |
| **Fath** | • | New-onset HF defined via ICD codes and/or HF exacerbation | Myocardial infarction by ICD-10/EHR coding | AKI/Acute renal failure | Reported as new-onset AF or AFL | NA | NA | NA |
| **Gongora** | • | HF and/or HF admission/exacerbation | NA | NA | NA | NA | Sepsis captured as safety endpoint (composite with neutropenic fever, EHR/clinical coding) | UTI captured as safety endpoint (EHR/clinical coding) |
| **Hendryx** | • | NA | NA | NA | NA | NA | NA | NA |
| **Henson** | • | NA | NA | AKI by ICD codes | Reported as new-onset AF or AFL | • | Sepsis captured by ICD-10 | • |
| **Huang** | • | NA | NA | NA | NA | NA | NA | NA |
| **Hwang** | • | HF hospitalization | Acute myocardial infarction as clinical outcome (ICD-10 claims coding, part of composite) | NA | NA | • | NA | NA |
| **Luo** | • | NA | NA | NA | NA | NA | NA | NA |
| **Perelman** | • | HF exacerbation/admission defined by hospitalization/ER visit (part of MACE composite) | MI captured within acute coronary syndrome (MACE composite, chart-review diagnosis) | NA | Not clearly reported | NA | NA | NA |

**Abbreviations:** HF = Heart Failure; MI = Myocardial Infarction; AF = Atrial Fibrillation; AFL = Atrial Flutter; AKI = Acute Kidney Injury ; EF ↓ = Left Ventricular Ejection Fraction reduction > 10 % from baseline; UTI = Urinary Tract Infections; NA = Not available; • = Endpoint present.

**Supplementary Table 3. PRISMA flowchart**


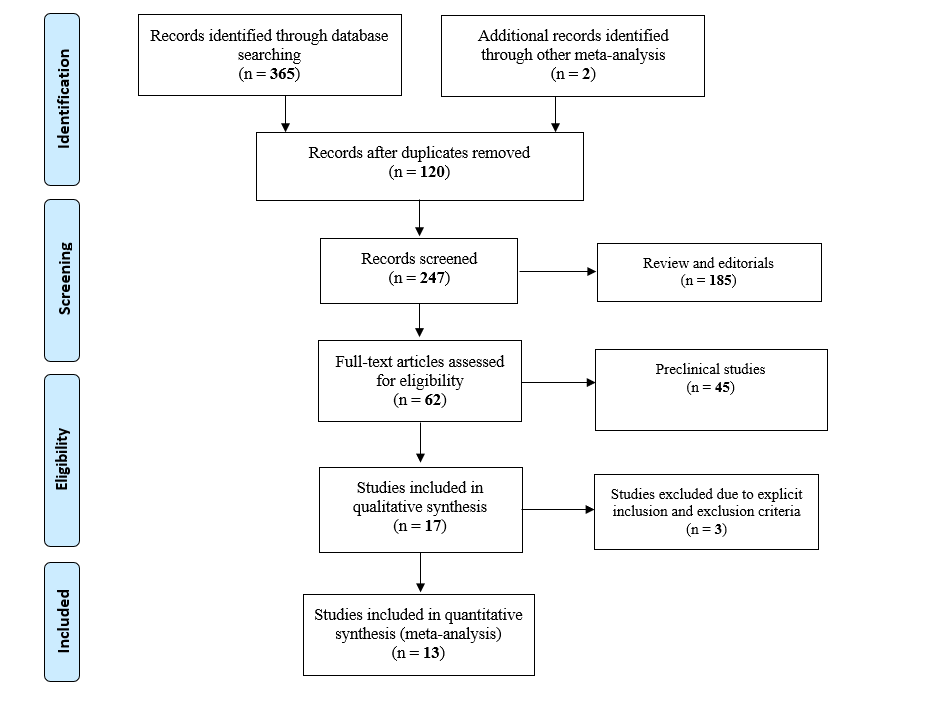


**Supplementary Table 4.1 Baseline features of the included studies.**

| **Study** | **Year** | **Study design** | **Type of cancer** | **Follow-up (months)** | **Mean age** | **Male** | **Smoking** | **Dyslipidemia** | **Hypertension** | **Beta -blockers** | **ACEi/**  **ARBs** | **Statins** |
| --- | --- | --- | --- | --- | --- | --- | --- | --- | --- | --- | --- | --- |
| **Abdel-Qadir et al.** | 2023 | Retrospective cohort study | Breast (49.5/32.7) hematologic/lymphoid malignancies (24.2/28.7) Other (26.3/38.6) | 19.2 | 71.0 | 37.8% | NA | 100.0% | 41.1% | 27.3% | 78.8% | 88.9% |
| **Avula et al.** | 2024 | Retrospective cohort study | Breast (15/1) Lymphomas (25/24) Myelodysplastic (40/34) GU (6/6) GI (18/22) Gynaecologic (3/3) Respiratory (6/5) Mesothelial (2/2) Neoplasms unspecified (22/22) Metastatic (30/29) | 24 | 67.6 | 58.4% | NA | 95.5% | 100.0% | 100.0% | NA | 24.0% |
| **Bhatti et al.** | 2024 | Retrospective cohort study | Breast (2273/2220) Lymphomas (1813/1683) Gastrointestinal (1683/1691) Myeloma/  MDS (1084/1224) Genitourinary (390/399) Gynecologic (599/642) Respiratory (312/304) Mesothelial/soft tissue (521/512) Metastatic (1440/1349) | 12 | 65.5 | 58.5% | NA | 63.7% | 72.9% | 39.0% | 53.4% | 59.3% |
| **Chiang et al. 2023** | 2023 | Retrospective propensity score–matched cohort study | Cancers: gastrointestinal (35%), genitourinary (18%), thoracic (13%), head & neck (10%), breast (11%), hematologic (5%), skin (2%), others (6%) | 18.8 | 65 | 53% | NA | 62% | 75% | 59% | 56% | 56% |
| **Chiang et al. 2024** | 2024 | Retrospective cohort study | Colorectal adenocarcinoma (100/100) | 33 | NA | NA | NA | NA | NA | NA | NA | NA |
| **Fath et al.** | 2024 | Retrospective cohort study | Haematological/lymphatic (37/40) Breast (26/25) GI (24/24) Female genital (9.3/8.8) Mesothelial (6.8/5.8) Urinary tract (5.0/4.5) | 24 | 62.5 | 48.0% | 11.0% | 68.5% | 77.5% | 55.5% | 45.0% | 71.5% |
| **Gongora et al.** | 2022 | Retrospective cohort study | Lymphoma (34/34) breast (28/23) genitourinary (9/19) gastrointestinal (16/7) sarcoma (6/7) leukaemia (3/3) others (3/6) | 18 | 60.0 | 50.0% | 32.5% | 57.0% | 63.0% | 29.5% | 45.0% | 58.5% |
| **Hendryx et al.** | 2022 | Retrospective cohort study | Hepatocellular carcinoma (100/100) | 20.4 | 74.8 | 68.3% | 92.8% | 100.0% | 43.8% | NA | NA | NA |
| **Henson et al.** | 2024 | Retrospective cohort study | Lymphoid/ hematologic (710/697) Breast (358/346) Genitourinary (74/77) Gastrointestinal (190/219) | 60 | 66.15 | 45.5% | 7.5% | 84.1% | 62.0% | 70.7% | 60.0% | 47.1% |
| **Huang et al.** | 2024 | Retrospective cohort study | Pancreatic (1/1) HCC (9/9) Oesophageal (1/1) Head/neck (12/12) Gastric (2/2) Lung (8/7) Colorectal (22/23) Gynaecologic (2/1) Breast (20/20) Prostate (9/9) Other (15/15) | 55.8 | NA | NA | NA | NA | NA | NA | NA | NA |
| **Hwang et al.** | 2023 | Retrospective cohort study | Lymphoma (12/15) Breast (61/47) GU (7/11) Other cancers (21/28) | 40.8 | NA | NA | NA | 48.0% | 46–53% (matched cohorts) | NA | NA | NA |
| **Luo et al.** | 2023 | Retrospective cohort study | Non-small cell lung cancer (100/100) | 21.2 | NA | NA | NA | NA | NA | NA | NA | NA |
| **Perelman et al.** | 2024 | Retrospective cohort study | NSCLC (21/25) Melanoma (8/18) RCC (25/22) HCC (25/18) Breast (17/2) Cervical (0/6) Other (4/8) | 28 | 71.0 | 62.0% | 8.0% | 34.0% | 59.0% | NA | NA | NA |

**Characteristics of the studies included.**

The table reports year of publication, study design, cancer type, use of propensity score matching (PSM), chemotherapy or other oncologic treatments, follow-up duration, mean age, sex distribution, smoking status, prevalence of dyslipidemia, hypertension, and diabetes, as well as concomitant cardiovascular therapies (beta-blockers, ACEi/ARBs, and statins).

**Abbreviations:** PSM = propensity score matching; GU = genitourinary; GI = gastrointestinal; MDS = myelodysplastic syndromes; NSCLC = non-small cell lung cancer; RCC = renal cell carcinoma; HCC = hepatocellular carcinoma; ICIs = immune checkpoint inhibitors; TKIs = tyrosine kinase inhibitors; VEGF = vascular endothelial growth factor; HER2 = human epidermal growth factor receptor 2; XELOX = capecitabine plus oxaliplatin; XELODA = capecitabine; FOLFIRI = folinic acid, fluorouracil, and irinotecan; FOLFOX = folinic acid, fluorouracil, and oxaliplatin.

**Supplementary Table 4.2 Study-level characteristics of included studies.**

| **Study** | **Overall Population** | **Diabetes subtype** | **SGLT2i agent** | **Timing of SGLT2i therapy** | **Active cancer** | **Race/ethnicity:** | **Pre-existing HF:** | **LVEF** | **Cancer therapy** | **Adjustment strategy and baseline covariates** |
| --- | --- | --- | --- | --- | --- | --- | --- | --- | --- | --- |
| Abdel-Qadir 2023 | Population-based administrative cohort (Canada); patients ≥65 years with treated diabetes undergoing anthracycline-based chemotherapy; n=933 (SGLT2i n=99, 10.6%); median follow-up 1.6 years | T2DM (T1DM not specified) | Dapagliflozin, empagliflozin, canagliflozin (no distribution reported) | Before/during chemotherapy (active use at chemotherapy initiation; prescription-based definition within 365 days prior) | Yes | Not reported | No | Not reported | Anthracyclines 100%, other therapies not detailed. | PS weighting (ATT) + Cox Covariates: age, sex, CV comorbidities, diabetes, medications, cancer type, cancer therapy |
| Avula 2024 | Retrospective cohort from TriNetX network; adult patients with T2DM, cancer, and exposure to cardiotoxic antineoplastic therapy, with subsequent CTRCD or HF; n=6,988 (PS-matched n=640 vs 640); follow-up 2 years | T2DM | Dapagliflozin, empagliflozin, canagliflozin (no distribution reported) | After cardiotoxic therapy / after CTRCD-HF diagnosis (timing relative to therapy not precisely defined) | History of cancer treated with cardiotoxic therapies; status not clearly defined | ~68% White, ~9% Hispanic | Included (population defined by CTRCD or HF after cancer therapy). | LVEF ≤40% in ~22% (SGLT2i) vs ~12% (pre-matching); ~21.6% vs ~19.8% post-matching. Not all patients had overt HF. | Mixed cardiotoxic therapies: anthracyclines (~19%), antimetabolites (fluorouracil ~35%), monoclonal antibodies (bevacizumab ~20%), alkylating agents (cyclophosphamide ~22%), TKIs, proteasome inhibitors, radiation (~11%) | PS matching (1:1, nearest neighbor, caliper 0.1) + Cox / OR models  Covariates: age, sex, race, comorbidities (HTN, CKD, AF), medications (including GDMT), cancer type, prior healthcare utilization |
| Bhatti 2024 | Retrospective cohort (TriNetX); adults ≥18 years with T2DM and cancer exposed to cardiotoxic therapies, without prior cardiomyopathy/HF; n=95,203 (PS-matched n=8,675 vs 8,675); mean age ~65 years; follow-up 12 months | T2DM | Empagliflozin, dapagliflozin, canagliflozin (no distribution reported; empagliflozin showed strongest association in subgroup analysis) | Before cancer therapy (baseline exposure prior to initiation of cardiotoxic therapy) | Yes | White ~71%, Black ~11%, Hispanic ~7–8% | No | Not reported | Mixed cardiotoxic therapies: anthracyclines ~24.5%, monoclonal antibodies ~21%, antimetabolites ~19%, TKIs ~17%, proteasome inhibitors ~8%, alkylating agents ~7%, aromatase inhibitors ~3% | PS matching (1:1, nearest neighbor, caliper 0.1) + Cox Covariates: age, sex, race, comorbidities (HTN, CKD, AF, IHD), diabetes severity (HbA1c), medications (GDMT), cancer type |
| Chiang 2023 | Retrospective propensity score-matched cohort; adults ≥18 years with T2DM and cancer; n=8,640 (PS-matched n=878 vs 878); median age 65 years; median follow-up ~18.8 months | T2DM | Empagliflozin (~49%), dapagliflozin (~38%), canagliflozin (minor proportion) | After cancer diagnosis (index date = first SGLT2i prescription post-cancer diagnosis; includes patients already on therapy but continued after diagnosis) | Yes | Asian | Included (~5% baseline HF in both groups after matching) | Not reported | Mixed: antimetabolites ~18%, platinum ~12%, plant alkaloids ~11%, anthracyclines ~8%, TKIs ~4%, immune checkpoint inhibitors ~1%, radiotherapy ~3% | PS matching (1:1, nearest neighbor, caliper 0.2 SD logit) + Cox + competing risk + time-varying Cox Covariates: age, sex, cancer type, metastatic disease, comorbidities, cardiovascular medications, cancer therapy, institution, year of diagnosis |
| Chiang 2024 | Retrospective cohort (2 tertiary centers, Taiwan); adults with T2DM and colorectal adenocarcinoma; n=1,347 screened, matched cohort n=92 vs 92; median age 68 years | T2DM | Canagliflozin, dapagliflozin, empagliflozin (no distribution reported) | After cancer diagnosis (index date = first SGLT2i prescription after cancer diagnosis; includes continuation of prior therapy; at least 14 days of post-diagnosis exposure required) | Yes | Asian | Included (baseline HF 9% in SGLT2i group vs 1% in non-SGLT2i group) | Not reported | Colorectal cancer-directed therapy: surgery 92%, FOLFOX 29%, tegafur/uracil 17%, FOLFIRI 11%, monoclonal antibodies 9%, XELODA 4%, XELOX 2%, radiotherapy 1% | Matching on age, sex, and cancer stage + multivariable Cox + time-varying Cox. Covariates: age, sex, ECOG-PS, cancer stage, chemotherapy regimen, surgery, radiotherapy, comorbidities, HbA1c, non-SGLT2i diabetes medications |
| Fath 2024 | Retrospective cohort (TriNetX); adults ≥18 years with cancer treated with anthracycline-based chemotherapy, without prior HF; n=79,074 screened, PS-matched n=706 vs 706; mean age 62.5 years | DM not specified (post-PSM ~90% had DM) | Empagliflozin 57%, canagliflozin 31%, dapagliflozin 26%, ertugliflozin 1.4 | During anthracycline therapy / at anthracycline initiation (exact timing not further specified) | Yes | White 61%, Black or African American 13%, Hispanic or Latino 11% | No | Yes (mean LVEF 62.8% vs 63.6% after PSM) | Anthracycline-based (100%) | PS matching (1:1, nearest neighbor, caliper 0.1) + Cox + Kaplan-Meier  Covariates: age, sex, race/ethnicity, cancer type, HTN, DM, dyslipidemia, IHD, cerebrovascular disease, AF/AFL, LVEF, CKD, ESRD, creatinine, OSA, COPD, smoking, alcohol disorders, anthracycline type, cardiovascular medications |
| Gongora et al. | Retrospective cohort (single academic center, USA); patients with DM and cancer treated with anthracyclines; n=128 (SGLT2i n=32 vs controls n=96; 3:1 matching); mean age ~60 years; median follow-up 1.5 years | DM not specified | Empagliflozin 50%, canagliflozin 34%, dapagliflozin 16% | Before/during anthracycline therapy (patients on SGLT2i prior to or during treatment; only those treated during anthracyclines included in final cohort) | Yes | Reported: White 91%, Black 6%, Asian 3% | Included (~6–7% baseline HF) | Yes (baseline LVEF ~62% vs 64%; available in ~77% of patients) | Anthracycline-based (100%) | Matching (3:1) on age, sex, and anthracycline start date; no PS Covariates: demographics, CV risk factors, comorbidities, medications, cancer type and stage, anthracycline dose, echocardiographic parameters |
| Hendryx 2022 | Retrospective cohort (SEER-Medicare, USA); patients ≥66 years with HCC and T2DM; n=3,185; mean age 74.8 years; mean follow-up ~20 months | T2DM | Canagliflozin, dapagliflozin, empagliflozin, ertugliflozin (no distribution reported) | Before and/or after cancer diagnosis (duration-based exposure; primary analyses emphasize use before diagnosis) | Yes | White ~51%, Black ~10%, Asian ~16%, Hispanic ~21% | No | Not reported | Mixed: surgery ~24%, chemotherapy ~40%, radiotherapy ~25% | Multivariable Cox Covariates: age, sex, race/ethnicity, marital status, CKD, HTN, CVD, diabetes duration, metformin use, HBV/HCV, alcohol-related disease, cirrhosis, cancer stage, cancer treatments |
| Henson 2024 | Retrospective cohort (TriNetX US Collaborative Network, 55 HCOs); patients with cancer, heart failure and prior anthracycline exposure; n=16,175 (SGLT2i: 691; no SGLT2i: 15,484) | Not explicitly reported. Likely predominantly T2DM given SGLT2i use | Not reported | Exact timing relative to chemotherapy not clearly specified | Yes | White 65%, Hispanic or Latino 5%, Black or African 20%, Unknown 16% | Yes | Not reported | Anthracycline-based (100%) | Propensity score matching (TriNetX built-in; details in PSM section) Covariates: matching on demographic,comorbidities, treatments |
| Huang 2024 | Retrospective cohort (Taiwan Cancer Registry linked to NHIRD); adults with T2DM and non-metastatic cancer receiving curative treatments; n=50,133 after propensity score matching (SGLT2i: 16,711; non-SGLT2i: 33,422) | T2DM | Not reported | After cancer diagnosis | Yes | Asian | No | Not reported | Curative treatments (surgery ± chemotherapy/radiotherapy; not further detailed) | Propensity score matching (2:1, caliper 0.1) + multivariable Cox regression  Covariates: age, sex, year of diagnosis, cancer type, cancer stage (AJCC), number and type of antidiabetic medications, diabetes severity (aDCSI), BMI, smoking, alcohol use, comorbidities, Charlson Comorbidity Index |
| Hwang 2023 | Retrospective nationwide cohort (Korean HIRA database); adults ≥18 years with newly diagnosed cancer undergoing anthracycline-containing chemotherapy; n=81,572 (T2DM with SGLT2i n=780; non-SGLT2i n=3,455; non-DM n=77,337); PS-matched cohorts constructed; mean follow-up ~3.4 years | T2DM | Not reported | At initiation of anthracycline therapy (exposure defined at index date; active use during chemotherapy) | Yes | Asian | No | Not reported | Anthracycline-based (100%); doxorubicin ~95%, epirubicin ~5%; high-dose anthracyclines ~28%; additional therapies: antimicrotubule agents ~44%, HER2 inhibitors ~15%, VEGF-targeting agents ~4%, alkylating agents ~5% | Propensity score matching (1:10 and 1:3 depending on cohort) + multivariable Cox  Covariates: age, sex, index year, hypertension, dyslipidemia, coronary artery disease, antithrombotics, statins, RAS inhibitors, beta-blockers, cancer type; additionally T2DM duration and number of oral hypoglycemic agents in T2DM comparison |
| Luo et al. 2023 | Retrospective cohort; patients ≥66 years with newly diagnosed NSCLC (2014–2017) and pre-existing diabetes; n=24,915; SGLT2i users n=531 (2.1%); mean follow-up ~21.2 months | Type 2 diabetes mellitus | Mixed class: canagliflozin, dapagliflozin, empagliflozin, ertugliflozin; predominant agent appears canagliflozin | Not explicitly linked to chemotherapy; exposure defined based on use after cancer diagnosis (time-varying duration; mean duration ~15.2 months) | Yes | Non-Hispanic White ~74.8%, Black ~11.0%, Asian/Pacific Islander ~6.5%, Hispanic ~6.9% | No | Not reported | Mixed NSCLC treatments: surgery ~23%, chemotherapy ~37.7%, radiation ~45%, immunotherapy ~4%, EGFR inhibitors ~3.9% | Multivariable Cox regression  Covariates: age, sex, race/ethnicity, marital status, CKD, hypertension, cardiovascular disease, cancer stage, histology, cancer treatments (surgery, chemotherapy, radiation, immunotherapy, EGFR inhibitors), diabetes duration, metformin use |
| Perelman 2024 | Retrospective, single-center cohort; cancer patients with T2DM treated with ICIs; n=119 (SGLT2i n=24, 20%); predominantly stage IV (88%); median follow-up 28 months | T2DM | Empagliflozin ~83%, dapagliflozin ~17% | Baseline therapy prior to ICIs initiation | Yes | Not reported | No | Not reported | ICIs: pembrolizumab 24%, nivolumab 8%, avelumab 8%, atezolizumab 30%, ipilimumab+nivolumab 30%; combination with chemotherapy/biologic therapy in ~33% (higher in SGLT2i: 67%) | Multivariable Cox regression  Covariates: age, sex, cancer type, cancer stage, treatment protocol (ICIs ± chemo/biologic), hypertension, dyslipidemia, ischemic heart disease, obesity, statin use, RAAS inhibitors |

**Abbreviations:** AF = atrial fibrillation; AFL = atrial flutter; AJCC = American Joint Committee on Cancer; ATT = average treatment effect on the treated; BMI = body mass index; CKD = chronic kidney disease; COPD = chronic obstructive pulmonary disease; CTRCD = cancer therapy–related cardiac dysfunction; CVD = cardiovascular disease; DM = diabetes mellitus; EGFR = epidermal growth factor receptor; ESRD = end-stage renal disease; GDMT = guideline-directed medical therapy; HCC = hepatocellular carcinoma; HF = heart failure; HFrEF = heart failure with reduced ejection fraction; HFpEF = heart failure with preserved ejection fraction; HCO = healthcare organization; HTN = hypertension; ICI = immune checkpoint inhibitor; IHD = ischemic heart disease; LVEF = left ventricular ejection fraction; NHIRD = National Health Insurance Research Database; NSCLC = non–small cell lung cancer; OSA = obstructive sleep apnea; PS = propensity score; PSM = propensity score matching; RAAS = renin–angiotensin–aldosterone system; SEER = Surveillance, Epidemiology, and End Results; SGLT2i = sodium–glucose cotransporter 2 inhibitor; T2DM = type 2 diabetes mellitus; TKI = tyrosine kinase inhibitor.

**Supplementary Table 5. Risk-of-bias assessment according to the ROBINS-I tool.**

| **Study (Year; Country/Setting)** | **D1 Confounding** | **D2 Selection** | **D3 Classification** | **D4 Deviations** | **D5 Missing** | **D6 Outcome measurement** | **D7 Reporting** | **Overall** |
| --- | --- | --- | --- | --- | --- | --- | --- | --- |
| **Abdel-Qadir et al.** (2023; Canada registry) | Moderate | Low | Low | Low | Low | Low | Low | Moderate |
| **Avula et al.** (2024; USA TriNetX registry) | Moderate | Moderate | Low | Low | Low | Moderate | Low | Moderate |
| **Bhatti et al.** (2024; USA TriNetX network) | Moderate | Moderate | Low | Low | Moderate | Moderate | Low | Moderate |
| **Chiang et al.** (2023; Taiwan, Heart) | Moderate | Low | Low | Low | Moderate | Low | Low | Moderate |
| **Chiang et al.** (2024; Taiwan, colorectal cancer cohort) | Moderate | Low | Low | Low | Moderate | Moderate | Low | Moderate |
| **Fath et al.** (2024; USA TriNetX network) | Moderate | Low | Low | Low | Moderate | Low | Low | Moderate |
| **Gongora et al.** (2022; USA, Mass General Brigham) | Moderate | Low | Low | Low | Moderate | Low | Low | Moderate |
| **Hendryx et al.** (2022, Texas A&M University, USA) | Moderate | Low | Moderate | Low | Low | Low | Low | Moderate |
| **Henson et al.** (2024; USA, TriNetX) | Moderate | Low | Low | Low | Moderate | Low | Low | Moderate |
| **Huang et al.** (2024; Taiwan Cancer Registry) | Moderate | Low | Low | Low | Moderate | Low | Low | Moderate |
| **Hwang et al.** (2023; South Korea, HIRA claims database) | Moderate | Low | Low | Low | Moderate | Low | Low | Moderate |
| **Luo et al.** (2023; USA, SEER–Medicare NSCLC) | Moderate | Low | Low | Low | Moderate | Low | Low | Moderate |
| **Perelman et al.** (2024; Israel, single-center ICIs cohort) | Moderate | Low | Low | Low | Moderate | Low | Low | Moderate |

**Supplementary Figures 1. Funnel plots and DOI Plots**


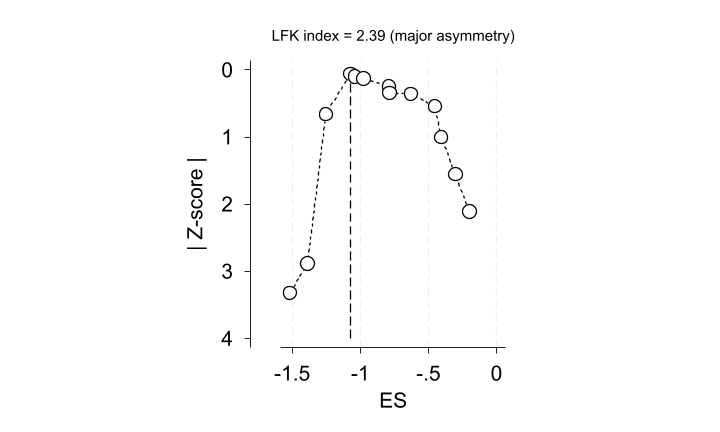
**1.1** **All-cause mortality**


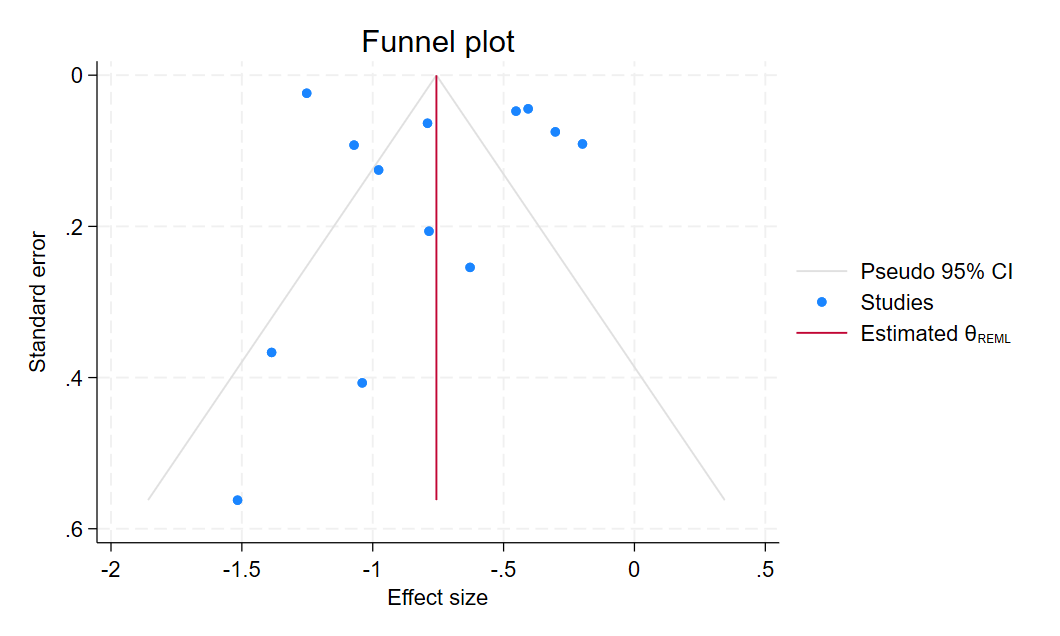


**1.2 HF composite**

**
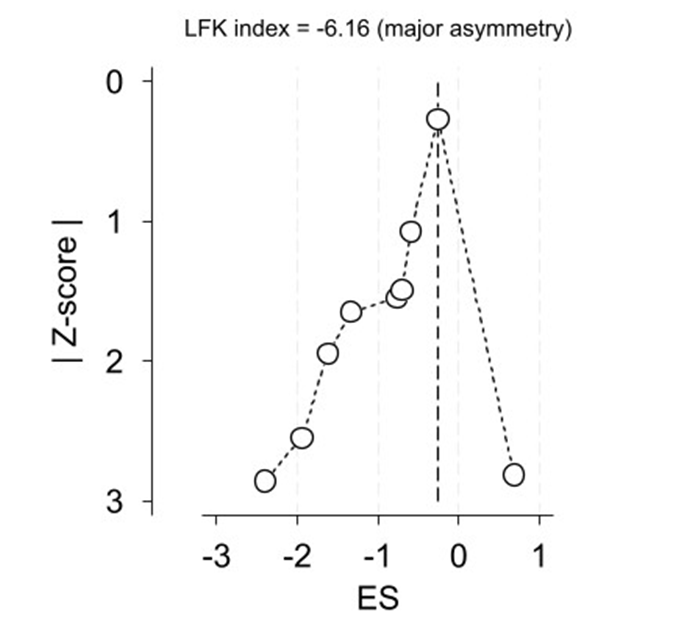

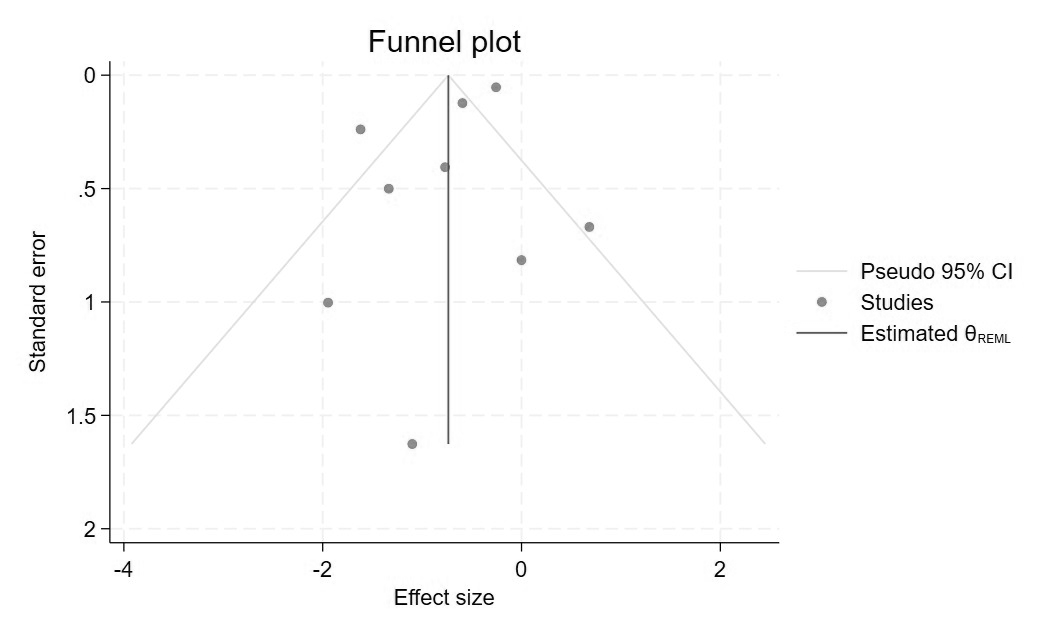
**

**1.3** **Atrial Fibrillation/Atrial Flutter**

**
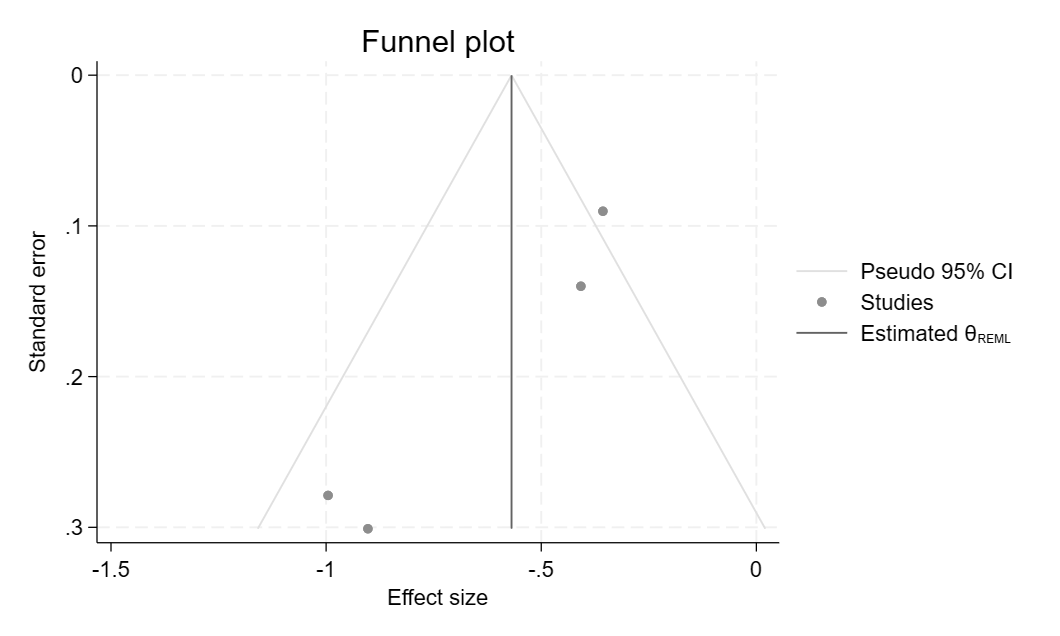
**

**1.4** **Myocardial Infarction**


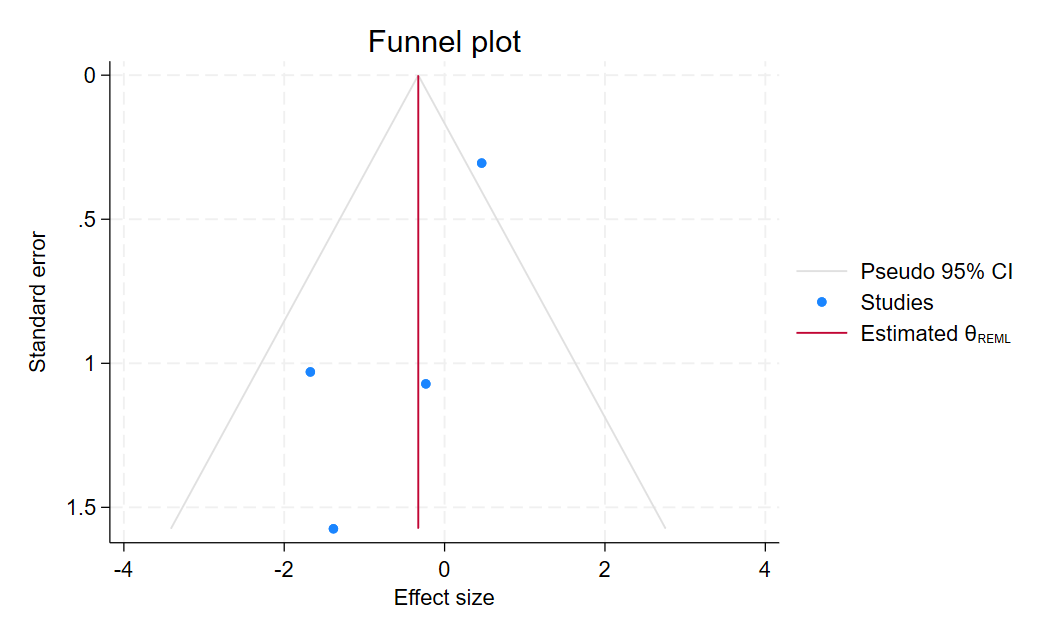


**1.5 Stroke**

**1.6** **Urinary Tract Infections**

**
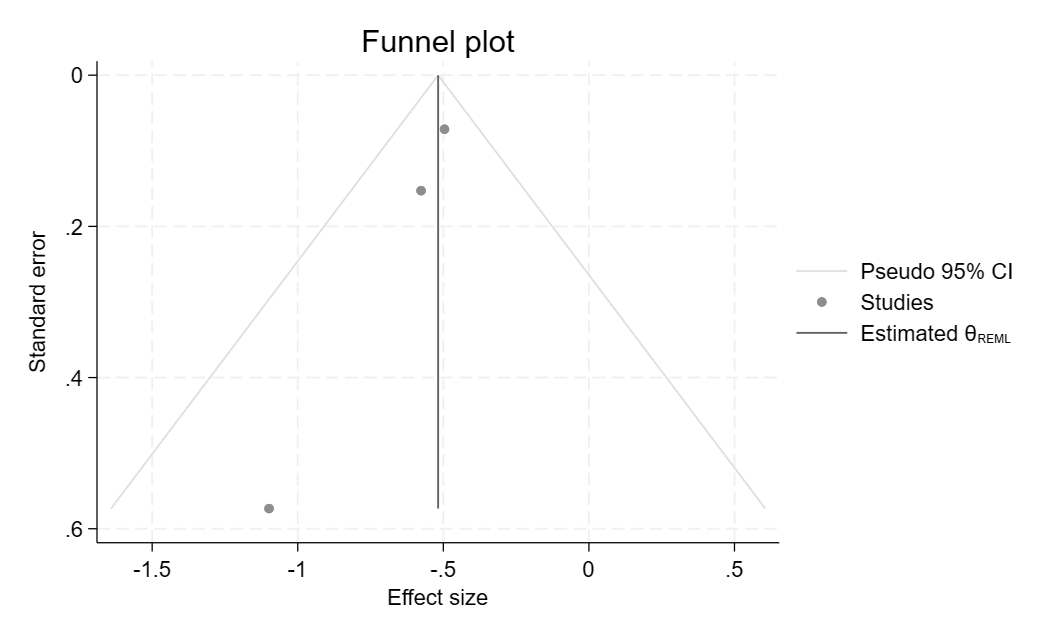
**

**1.7 Sepsis**

**1.8** **Acute Kidney Injury**

**Supplementary Figures 2. Sensitivity analysises.**

**2.1** **All-Cause Mortality - sensitivity analysis anthracyclines**

**2.2** **HF Composite – sensitivity analysis anthracyclines**

**2.3 Univariable meta-regression (metareg, REML, Knapp–Hartung)**

**Endpoint:** All-cause mortality

| **Moderator** | **Coefficient** | **95% CI** | **p value** | **RR per +10 units** |
| --- | --- | --- | --- | --- |
| Percent Dyslipidemia (+10%) | 0.0634 | -0.2141 ; 0.3409 | 0.606 | 1.07 |
| Percent Hypertension (+10%) | 0.0264 | -0.0859 ; 0.1388 | 0.612 | 1.03 |
| Percent Breast Cancer (+10%) | 0.0998 | -0.0749 ; 0.2745 | 0.228 | 1.10 |
| Percent GI Cancer (+10%) | 0.0064 | -0.1173 ; 0.1301 | 0.903 | 1.01 |
| Percent Hematologic Cancer (+10%) | 0.1105 | -0.0666 ; 0.2877 | 0.178 | 1.12 |
| Follow-up duration (+10 months) | -0.0969 | -0.2434 ; 0.0496 | 0.173 | 0.91 |

**2.4 Univariable meta-regression (metareg, REML, Knapp–Hartung)**

**Endpoint:** HF composite

| **Moderator** | **Coefficient** | **95% CI** | **p value** | **RR per +10 units** |
| --- | --- | --- | --- | --- |
| Percent Dyslipidemia (+10%) | -0.0207 | -0.0609 to 0.0196 | 0.31 | 0.81 |
| Percent Hypertension (+10%) | -0.0037 | -0.0251 to 0.0178 | 0.74 | 0.96 |
| Percent Breast Cancer (+10%) | 0.0010 | -0.0351 to 0.0371 | 0.96 | 1.01 |
| Percent GI Cancer (+10%) | -0.0068 | -0.0468 to 0.0332 | 0.74 | 0.93 |
| Percent Hematologic Cancer (+10%) | -0.0062 | -0.0556 to 0.0432 | 0.81 | 0.94 |
| Follow-up duration (+10 months) | 0.0207 | -0.0553 to 0.0968 | 0.59 | 1.23 |

**2.5 Forest plot of confounding-adjusted hazard ratios for all-cause mortality.**

**
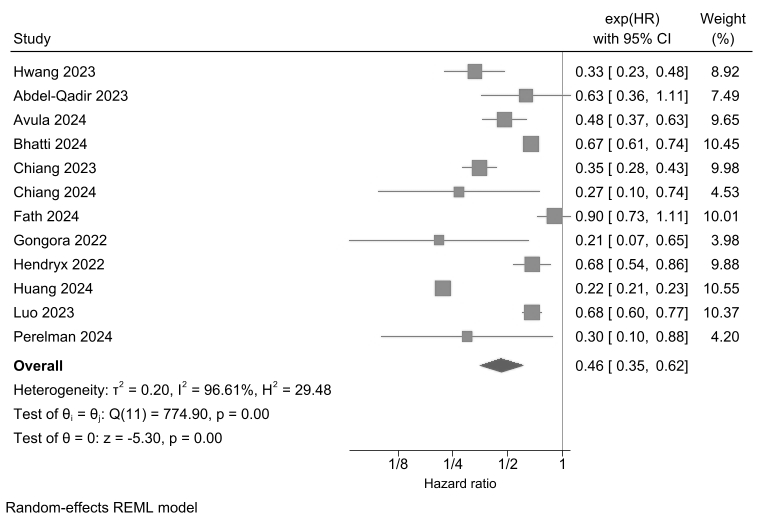
**

**2.6 Sensitivity analysis excluding potential overlapping cohorts – All Cause Mortality**

**
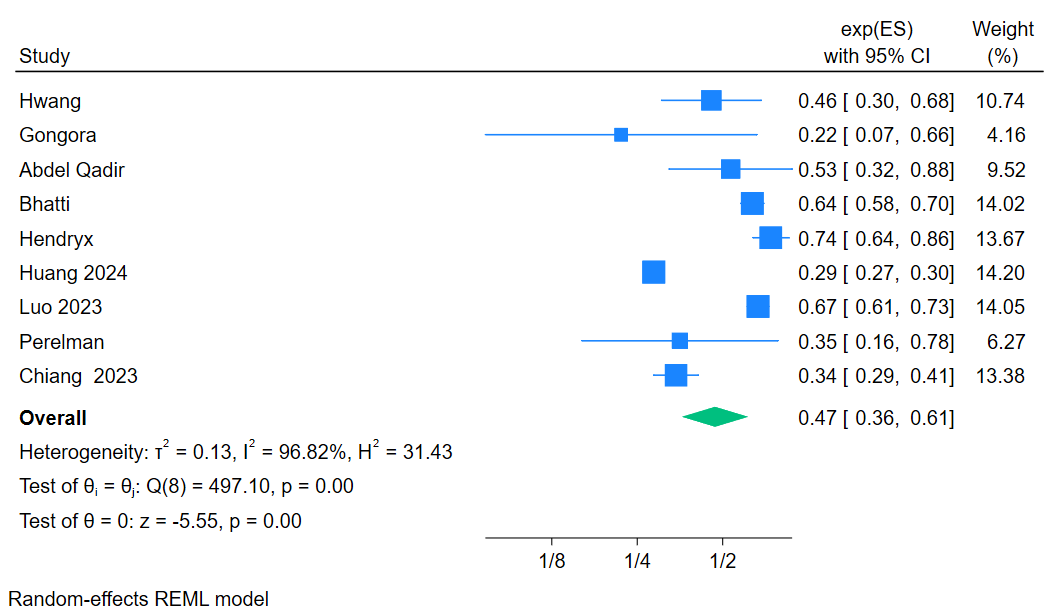
**

**2.7 Sensitivity analysis excluding potential overlapping cohorts – HF composite**

**
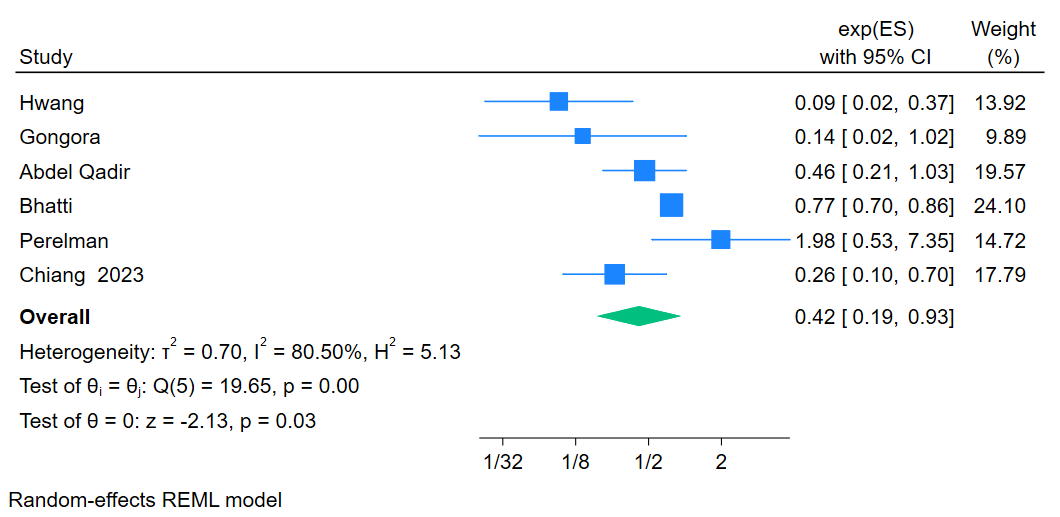
**

**2.8 Sensitivity analysis focusing on HF hospitalizations only**

**
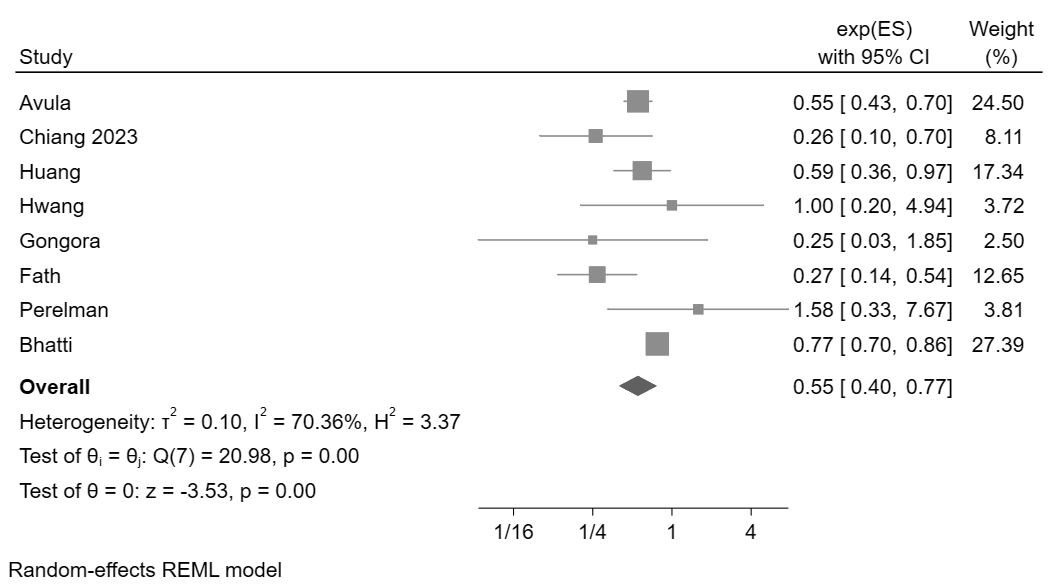
**
